# Isolation and Evaluation of the Gut-Associated Bacterium *Staphylococcus gallinarum* KI4: Effects on Survival and Cocoon Traits of *Bombyx mori* Under *Bacillus cereus* Challenge

**DOI:** 10.3390/insects17101075

**Published:** 2026-10-17

**Authors:** Olle Terenius, Siripuk Suraporn

**Affiliations:** 1 Department of Cell and Molecular Biology, Uppsala University, 752 37 Uppsala, Sweden; olle.terenius@icm.uu.se; 2 Department of Biology, Faculty of Science, Mahasarakham University, Kantarawichai District, Maha Sarakham 44150, Thailand

**Keywords:** *Bombyx mori*, sericulture, resource insect, gut-associated bacteria, *Staphylococcus gallinarum*, *Bacillus cereus*, bacterial antagonism, host protection

## Abstract

Indigenous insect-associated bacteria may provide biological resources for improving disease resilience in insect production. We isolated and functionally screened gut-associated bacteria from the Thai polyvoltine Nang Lai strain of *Bombyx mori*. Fifty colonies were recovered following alkaline enrichment. Partial 16S rRNA gene sequence analysis and phylogenetic comparison assigned isolates KI1, KI2, KI3, KI4, and KI9 to *Staphylococcus gallinarum* and KI6 to *Enterococcus mundtii*; the sequences were deposited in GenBank under accession numbers PZ319142-PZ319147. KI2 and KI4 produced inhibition zones against *Bacillus cereus*, *Bacillus thuringiensis*, and *Serratia marcescens*, whereas KI6 and KI9 showed no detectable inhibition. KI4 was selected for in vivo evaluation because it produced the largest inhibition zone against *B. cereus* (18.0 ± 1.1 mm). Under non-challenged conditions, KI4 supplementation did not differ significantly from the untreated control in final larval weight, cocooning, pupation, fresh cocoon weight, or shell weight. *B. cereus* challenge reduced endpoint survival to 49.33 ± 2.31%, whereas daily KI4 supplementation initiated before challenge and continued thereafter was associated with higher endpoint survival (77.33 ± 2.31%) and higher cocooning and pupation than the pathogen-only treatment. The in vitro antagonism and in vivo host response represent separate observations because a non-antagonistic bacterial control was not included. These findings support further evaluation of *S. gallinarum* KI4 (GenBank accession no. PZ319145) as a candidate host-derived bacterial resource for sericulture. Intestinal persistence, the mechanism and specificity of the host response, biosafety, and field efficacy require further investigation before practical application.

## Simple Summary

The silkworm, *Bombyx mori*, is an economically important insect whose survival can be severely affected by bacterial disease. This study screened bacteria recovered from the gut of the Thai polyvoltine Nang Lai strain to identify a host-derived isolate with potential use in sericulture. Five representative isolates were assigned to *Staphylococcus gallinarum* and one isolate to *Enterococcus mundtii* based on partial 16S rRNA gene sequence analysis and phylogenetic comparison. Two *S. gallinarum* isolates inhibited bacterial pathogens of silkworms in laboratory assays. Isolate KI4 was selected for a feeding experiment because it showed the largest inhibition zone against *Bacillus cereus*. Under non-challenged conditions, KI4 supplementation alone did not produce statistically significant differences from the untreated control in final larval weight, cocooning, pupation, fresh cocoon weight, or shell weight. During *B. cereus* challenge, larvae receiving continued KI4 supplementation had higher endpoint survival, cocooning, and pupation than larvae challenged with *B. cereus* alone. These findings identify KI4 as a candidate host-derived bacterial resource for further evaluation in sericulture. The study does not establish intestinal colonization, a specific protective mechanism, or comprehensive safety; these questions, together with controlled validation and field testing, require further investigation.

## 1. Introduction

Insects used for silk, food, feed, pollination, and biological conversion are increasingly regarded as production resources whose value depends on stable growth, efficient bioconversion, and effective disease management. In this context, the gut microbiota is relevant because resident microorganisms can participate in nutrient use, microbial competition, community stability, and responses to environmental or infectious stress [1,2,3,4,5,6,7,8]. Recent studies of resource insects have therefore combined production traits with microbiological information to identify interventions that improve insect performance [9,10,11,12,13,14,15]. However, taxonomic identification alone does not establish biological function. Candidate microorganisms must be isolated, characterized, and subjected to functional screening appropriate to the intended host and use [12,13,14,15,16].

Gut-associated bacteria can restrict other microorganisms through competition for nutrients and attachment sites or through the production of organic acids, hydrogen peroxide, bacteriocin-like substances, and other inhibitory metabolites [6,17,18,19]. Protective effects of host-derived bacteria have been reported in several insect models. For example, an indigenous *Enterococcus mundtii* isolate increased resistance of *Tribolium castaneum* to *Bacillus thuringiensis*, a host-derived *Pediococcus pentosaceus* strain influenced growth and survival of *Tenebrio molitor*, and lactic acid bacteria reduced pesticide-associated stress in honey bees [9,10,11]. In silkworms, recent studies with *P. pentosaceus*, *Bacillus subtilis*, and *Lactobacillus* spp. have shown strain-specific effects on growth, cocoon traits, gut microbiota, metabolism, and/or resistance to pathogenic challenge [12,13,14,15]. Accordingly, KI4 is described here as a candidate host-derived bacterial resource rather than as a probiotic, because accepted probiotic terminology requires defined identity, demonstrated host benefit, and assessment for the intended use [20,21].

The domesticated silkworm, *Bombyx mori*, is both a major economic insect and a model for studies of insect physiology, immunity, and host–microbe relationships [5,22,23,24]. Intensive rearing can facilitate bacterial disease, and pathogens such as *Bacillus cereus*, *B. thuringiensis*, and *Serratia marcescens* can reduce larval survival and cocoon production [25,26,27,28,29]. Biological approaches based on bacteria originating from the silkworm itself may complement hygienic management, provided that the isolates are accurately identified, show no evident adverse effects on the host, and retain a measurable protective phenotype in vivo. Evidence of inhibition in vitro therefore warrants a separate evaluation of host outcomes under pathogen challenge.

This study was designed as an initial, resource-oriented evaluation of a host-derived bacterial candidate for sericulture, rather than as a mechanistic investigation of host–pathogen interactions or a demonstration of intestinal colonization. The silkworm midgut is strongly alkaline, and this physicochemical environment can be used as a selective criterion for recovering culturable bacteria tolerant of alkaline conditions relevant to the host gut. Because a candidate intended for practical sericulture development must be recoverable and propagated reproducibly, cultivability was an intentional screening criterion. The present study therefore used alkaline enrichment as a targeted screening approach rather than as a comprehensive survey of the gut microbiota. Our objectives were to (i) identify representative isolates by partial 16S rRNA gene analysis and phylogenetic comparison, (ii) compare antibiotic-response profiles, solvent-dependent cell-surface partitioning, and in vitro antagonistic activity, and (iii) evaluate the effects of the selected isolate KI4 on growth, survival, and cocoon traits during oral *B. cereus* challenge. Accordingly, the study links isolate-level screening with a host-level challenge outcome and was not intended to provide a comprehensive microbiome or mechanistic analysis.

## 2. Materials and Methods

### 2.1. Silkworm Strain and Rearing

The Thai polyvoltine Nang Lai (NL) strain of *B. mori* was maintained in the Silkworm Rearing Laboratory, Department of Biology, Mahasarakham University, Thailand. Eggs were incubated at 26–27 °C for approximately 8 days. Before hatching, eggs were surface-disinfected by immersion in 70% ethanol for 2 min followed by 3% formalin for 8 min, rinsed thoroughly with sterile distilled water, briefly immersed again in 70% ethanol, and air-dried under sterile conditions. Larvae were reared at 25–28 °C and 70–80% relative humidity under a natural photoperiod and were supplied with fresh mulberry leaves three times daily.

### 2.2. Isolation of Indigenous Gut-Associated Bacteria

Fifth-instar larvae on day 4 were selected for dissection. Each larva was surface-disinfected with 70% ethanol for 2 min, rinsed twice with sterile distilled water, and air-dried. Twenty midguts were aseptically dissected and combined as one pooled isolation sample. The pooled tissue was homogenized in 90 mL of sterile saline using a sterile mortar and pestle. The homogenate was inoculated into de Man, Rogosa and Sharpe (MRS) broth (HiMedia Laboratories Pvt. Ltd., Thane, India) adjusted to pH 10.0 and incubated at 37 °C for 2 h. This enrichment was used as a selective recovery step consistent with the alkaline physicochemical environment of the silkworm midgut; it was not intended to recover the complete culturable community. Serial dilutions (10^−1^–10^−4^) were spread onto MRS agar supplemented with CaCO_3_ and 0.004% bromocresol purple. Plates were incubated anaerobically at 37 °C for 48 h using an AnaeroPack system (Mitsubishi Gas Chemical Co., Tokyo, Japan). Colonies exhibiting similar morphological characteristics and surrounding clear zones were selected and purified by repeated streaking.

### 2.3. Morphological Characterization

Colony shape, margin, elevation, surface appearance, opacity, and approximate diameter were recorded from purified cultures. Gram staining was performed using crystal violet, Gram iodine, ethanol decolorization, and safranin counterstaining. Cell morphology and arrangement were examined at 1000× magnification. Colony and microscopic images were recorded as visual documentation. Ten isolates exhibiting similar colony morphology and Gram-positive coccoid cells were selected for partial 16S rRNA gene sequencing. Taxonomic assignment was based on sequence analysis and phylogenetic comparison rather than on morphology alone.

### 2.4. Partial 16S rRNA Gene Sequencing and Phylogenetic Analysis

Genomic DNA was extracted from overnight bacterial cultures using the DNeasy Blood and Tissue Kit (QIAGENGmbH, Hilden, Germany) according to the manufacturer’s instructions. The 16S rRNA gene was amplified using primers 27F (5′-AGAGTTTGATCMTGGCTCAG-3′) and 1492R (5′-TACGGYTACCTTGTTACGACTT-3′). Each 25 µL PCR reaction contained 1× PCR buffer, 2.5 mM MgCl_2_, 0.2 mM dNTPs, 0.2 µM of each primer, 1 U Taq DNA polymerase, and approximately 50 ng of template DNA. PCR cycling conditions were 95 °C for 5 min; 35 cycles of 95 °C for 30 s, 55 °C for 30 s, and 72 °C for 90 s; followed by a final extension at 72 °C for 10 min. Amplicons were examined by electrophoresis on 1.5% agarose gels using a GeneDireX OneMARK 100 DNA ladder (GeneDireX, Inc., Taoyuan, Taiwan; Cat. No. DM101-0100), purified using the QIAquick PCR Purification Kit (QIAGEN, Germany), and sequenced bidirectionally by Macrogen Inc. (Seoul, Republic of South Korea). Forward and reverse reads were quality-checked, trimmed, assembled into consensus sequences, and compared with GenBank records using NCBI nucleotide BLAST (BLASTn; web-based interface).

The sequences were deposited in GenBank under accession numbers PZ319142–PZ319147.

For phylogenetic analysis, the six 16S rRNA gene sequences obtained in this study were analyzed together with seven selected reference sequences. Evolutionary analyses were conducted in MEGA12 [30]. The phylogeny was inferred using the Maximum Likelihood method with the Tamura–Nei nucleotide substitution model [31]. Branch support was assessed using 1000 bootstrap replicates [32]. For the heuristic search, the initial tree was selected by comparing a Neighbor-Joining (NJ) tree [33] with a Maximum-Parsimony (MP) tree and retaining the starting tree with the superior log likelihood. The NJ tree was generated from a matrix of pairwise distances calculated using the Tamura–Nei model. The MP starting tree was the shortest tree obtained among 10 searches, each initiated from a randomly generated starting tree. The tree with the highest log likelihood (−3863.96) was retained. The complete multiple sequence alignment used for phylogenetic analysis, comprising 13 nucleotide sequences and 1871 aligned positions, is provided as Supplementary File S3. Alignment gaps are represented by hyphens (‘-’) and ambiguous nucleotide positions by N in Supplementary File S3. The original multiple sequence alignment algorithm and the MEGA12 settings for handling gaps and missing data during phylogenetic inference were not retained in the available analysis records; consequently, these settings could not be verified retrospectively.

### 2.5. Antibiotic Disk-Diffusion Profiles

Antibiotic-response profiles of the bacterial isolates were examined using the Kirby–Bauer disk-diffusion method [34] with commercial antibiotic discs (HiMedia, Maharashtra, India). Seven antibiotics were tested: chloramphenicol (30 µg), penicillin (10 units), tetracycline (30 µg), gentamicin (10 µg), amoxicillin (30 µg), streptomycin (10 µg), and erythromycin (15 µg). Overnight cultures were spread on Mueller–Hinton agar, antibiotic discs were applied, and plates were incubated at 37 °C for 24 h. Inhibition-zone diameters were measured in millimeters. Because taxon-specific interpretive breakpoints and full quality-control validation were not available for all isolates under the study conditions, the measurements were used only for comparative phenotypic characterization and are reported descriptively as inhibition-zone diameters; susceptible, intermediate, and resistant categories were not assigned.

### 2.6. Solvent-Dependent Cell-Surface Partitioning

Solvent-dependent cell-surface partitioning was assessed using a microbial adhesion-to-solvent assay [35,36]. Overnight cultures were harvested by centrifugation at 5000× *g* for 10 min at 4 °C, washed twice with phosphate-buffered saline (PBS, pH 7.2), and adjusted to an OD600 of 0.5. Equal volumes of bacterial suspension and each solvent (ethyl acetate or chloroform) were mixed separately and vigorously for 2 min and then allowed to separate. The OD600 of the aqueous phase was measured, and solvent affinity was calculated as [(ODinitial − ODfinal)/ODinitial] × 100. The resulting values were interpreted as solvent-dependent partitioning or affinity, rather than as a direct measure of intestinal adhesion. The assay was used only for comparative characterization of in vitro cell-surface properties and was not considered evidence of epithelial adhesion, intestinal persistence, or colonization [37,38,39,40]. This assay was performed on the subset KI2, KI4, KI6, and KI9; KI1 and KI3 were not included. The archived laboratory record does not document the original rationale for this subset selection, so these measurements are not presented as a complete comparison of all six sequenced isolates.

### 2.7. *In Vitro* Antagonistic Screening

Antagonistic activity against *Bacillus cereus*, *Bacillus thuringiensis*, and *Serratia marcescens* was evaluated using an agar-well diffusion assay [41]. Each indicator bacterium was adjusted to approximately 1.73 × 10^8^ CFU/mL and spread onto nutrient agar. Wells 6 mm in diameter were cut aseptically and filled with 100 µL of the test bacterial culture. Sterile MRS broth served as the negative control. Plates were incubated at 30 °C for 24 h. The reported value was the total diameter of the clear zone, including the 6-mm well. Assays were performed in triplicate; the reported *n* = 3 refers to replicate assay plates rather than independent isolate-recovery events. Antagonistic screening was performed on KI2, KI4, KI6, and KI9; KI1 and KI3 were not included. The archived laboratory record does not document the original rationale for selecting this subset. Archived records identify the indicator bacteria at the species level only; strain accession or source identifiers were not retained, and no such identifiers are inferred here.

### 2.8. Preparation of KI4 and Bacillus cereus for the Feeding Experiment

*Staphylococcus gallinarum* KI4 was cultured in de Man, Rogosa and Sharpe (MRS) broth at 37 °C for 24 h. Cells were harvested by centrifugation, washed twice with sterile phosphate-buffered saline (PBS, pH 7.2), and resuspended in sterile PBS. The bacterial concentration was determined by viable plate counting and adjusted to approximately 1 × 10^8^ CFU/mL for the feeding treatment. *Bacillus cereus* was cultured in nutrient broth at 30 °C for 24 h, harvested by centrifugation, washed with sterile PBS, and resuspended to a final concentration of 1.73 × 10^4^ CFU/mL for oral challenge. Thus, the in vivo KI4 preparation consisted of washed cells in PBS, whereas the agar-well antagonism assay used whole bacterial cultures. The archived laboratory record available for this revision did not retain a strain accession/source identifier or the original strain-identification workflow for the *B. cereus* challenge culture; therefore, no additional strain-level characterization is inferred.

### 2.9. Feeding Design

Larvae were randomly allocated to four treatment groups: G1, untreated control; G2, *S. gallinarum* KI4 supplementation; G3, *B. cereus* challenge; and G4, *S. gallinarum* KI4 supplementation + *B. cereus* challenge. Each group consisted of three independently maintained rearing replicates with 25 larvae initially allocated to each replicate (N = 75 per group). Each rearing replicate was treated as the experimental unit. The G1–G2 and G3–G4 contrasts were used to describe KI4-associated outcomes without and with pathogen challenge, respectively.

KI4 supplementation in G2 and G4 was initiated on day 2 of the third instar and continued once daily in the morning throughout the experimental period. For each replicate, 0.5 mL of KI4 suspension (approximately 1 × 10^8^ CFU/mL) was applied to fresh mulberry leaves provided as part of the normal feeding regimen. The KI4-treated leaves were air-dried for 5 min before being provided to the larvae. Subsequent feedings consisted of untreated fresh mulberry leaves. Larvae were fed three times daily throughout the larval rearing period. The stated concentration and volume represent the nominal inoculum applied to the leaves; the quantity actually ingested by individual larvae was not directly measured.

On day 2 of the fourth instar, larvae in G3 and G4 were fasted for 4 h before receiving a single oral challenge with *B. cereus*. For each replicate of 25 larvae, five fresh mulberry leaf pieces (2 × 2 cm each) were treated with a total of 0.5 mL of *B. cereus* suspension (1.73 × 10^4^ CFU/mL) and air-dried for 5 min before feeding. *B. cereus* was administered only once. After the challenge, larvae were returned to the normal feeding regimen, and KI4 supplementation in G4 was continued once daily in the morning until the experimental endpoint. Larvae in G1 received untreated mulberry leaves throughout the experiment, and G2 received KI4-treated leaves without *B. cereus* challenge. G1 and G2 were not subjected to the 4-h pre-challenge fasting procedure, and fully matched PBS-treated vehicle and fasting controls were not included. Accordingly, the design does not isolate possible effects of vehicle treatment or fasting across all four groups. No non-antagonistic bacterial supplementation group (e.g., KI9 or KI6) was included; therefore, the G3-versus-G4 comparison cannot distinguish a KI4-specific antagonistic effect from a nonspecific effect of bacterial supplementation.

Mortality was monitored until the developmental endpoint at the end of the fifth instar, immediately before mounting. Endpoint survival was calculated for each replicate as the number of larvae remaining alive divided by the initial denominator of 25 larvae. Cocooning and pupation were likewise recorded as counts and percentages relative to the initial 25 larvae per replicate. At the same developmental endpoint, total final larval weight was recorded for each replicate, and final larval weight per larva was calculated by dividing the total final larval weight by the number of surviving larvae in that replicate. Fresh cocoon weight and cocoon shell weight were subsequently summarized to obtain one replicate-level value for each independent rearing replicate. The underlying counts and replicate-level values are provided in Supplementary Table S1. The archived record does not preserve a formal mortality-check frequency, quantitative leaf-consumption measurements, the exact cocoon age at weighing, or a separate shell-preparation protocol; these details are therefore not reconstructed or inferred in the revised manuscript.

### 2.10. Statistical Analysis

Quantitative assays were performed with three replicates, as specified for each experiment. Results are presented as mean ± standard deviation (SD), where applicable. For the feeding experiment, the independent rearing replicate was the experimental unit (*n* = 3 per treatment). Final larval weight, cocooning percentage, pupation percentage, fresh cocoon weight, shell weight, and endpoint survival percentage were analyzed from replicate-level values using one-way analysis of variance followed by Tukey’s HSD multiple-comparison test in IBM SPSS Statistics version 23.0 (IBM Corp., Armonk, NY, USA), with *p* < 0.05 considered statistically significant. Survival, cocooning, and pupation are bounded percentage outcomes, and some treatment groups had zero observed between-replicate variance; therefore, these inferential results are interpreted conservatively and are presented together with the underlying counts and replicate-level values in Supplementary Table S1. The endpoint was the end of the fifth instar immediately before mounting and was analyzed as a developmental endpoint rather than as individual time-to-event data; no Kaplan–Meier or hazard analysis was performed. Holm–Bonferroni adjustment was applied to the five omnibus ANOVA *p*-values for growth performance and cocoon traits (familywise α = 0.05; see Supplementary File S2, Holm–Bonferroni worksheet). Endpoint survival was not counted as a sixth test because its replicate-level percentages were identical to cocooning. Tukey HSD adjusts within-outcome pairs only; equal denominators do not verify ANOVA assumptions, so all inferences, including shell weight, are exploratory.

## 3. Results

### 3.1. Isolation and Morphology of Indigenous Gut-Associated Bacteria

**Figure 1:**
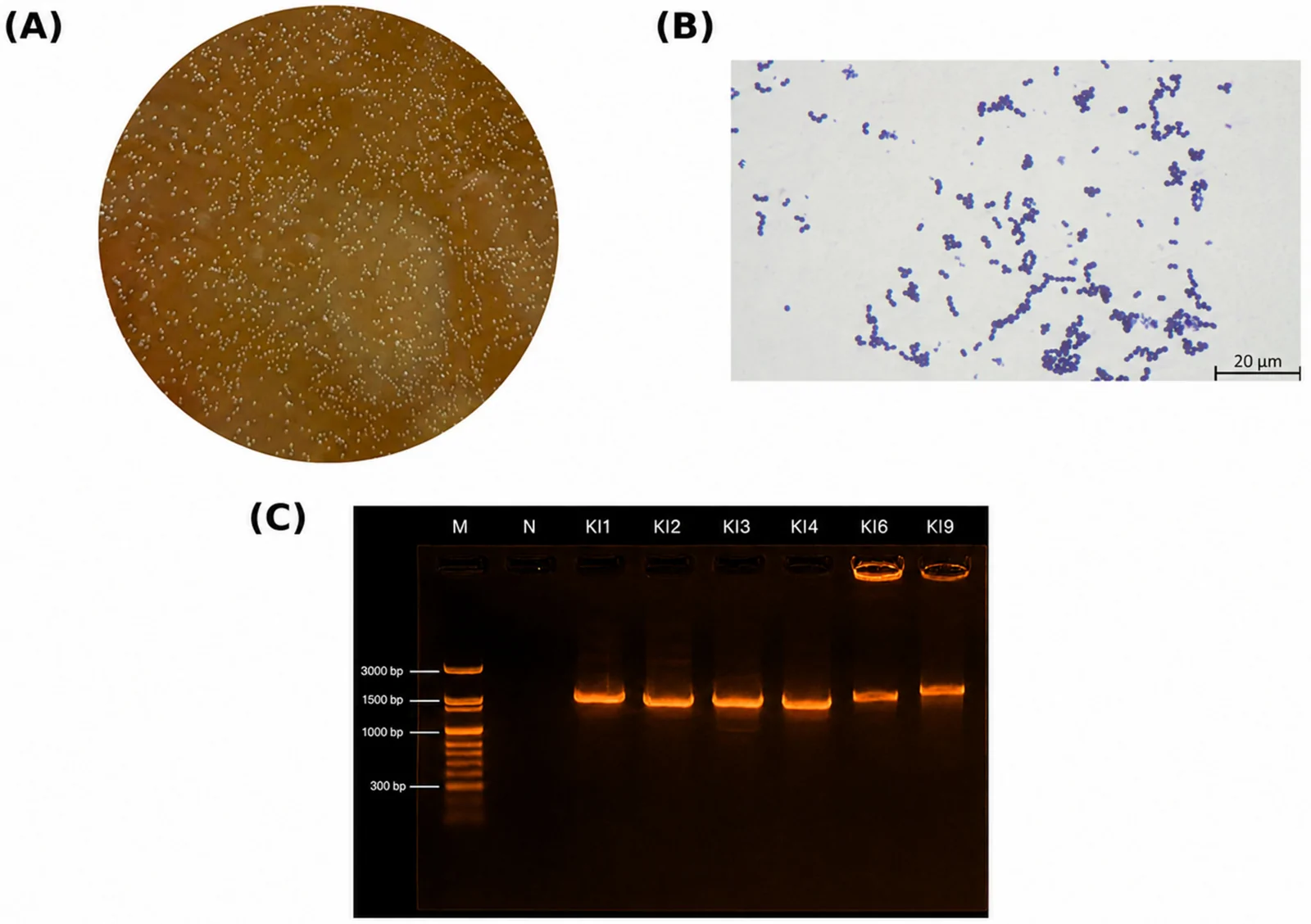
Representative isolation and characterization of indigenous gut-associated bacteria from *Bombyx mori*. (**A**) Colonies recovered on MRS-CaCO_3_ agar following alkaline enrichment; (**B**) Gram-stained cells of isolate KI4 observed under a light microscope using a 100× oil-immersion objective, showing Gram-positive cocci arranged predominantly in clusters (scale bar = 20 µm); and (**C**) agarose gel electrophoresis of 16S rRNA gene PCR products. M, GeneDireX OneMARK 100 DNA Ladder; N, negative control; KI1, KI2, KI3, KI4, KI6, and KI9, bacterial isolates.

A total of 50 colonies were recovered from the pooled midgut sample after alkaline enrichment. Colonies selected for purification were generally circular, smooth, convex, and cream to opaque white, with diameters of approximately 1–2 mm. The purified isolates were Gram-positive cocci arranged predominantly in irregular clusters. Based on the morphological and Gram-staining criteria described above, ten isolates were selected for partial 16S rRNA gene sequencing. Sequencing of four isolates yielded ambiguous chromatograms; these isolates were excluded, leaving six isolates for molecular and functional comparison. Representative colony morphology, Gram-stained cells, and partial 16S rRNA gene amplicons are shown in Figure 1. The expected amplicon size was approximately 1.5 kb.

### 3.2. Molecular Identification and Phylogenetic Analysis

**Figure 2:**
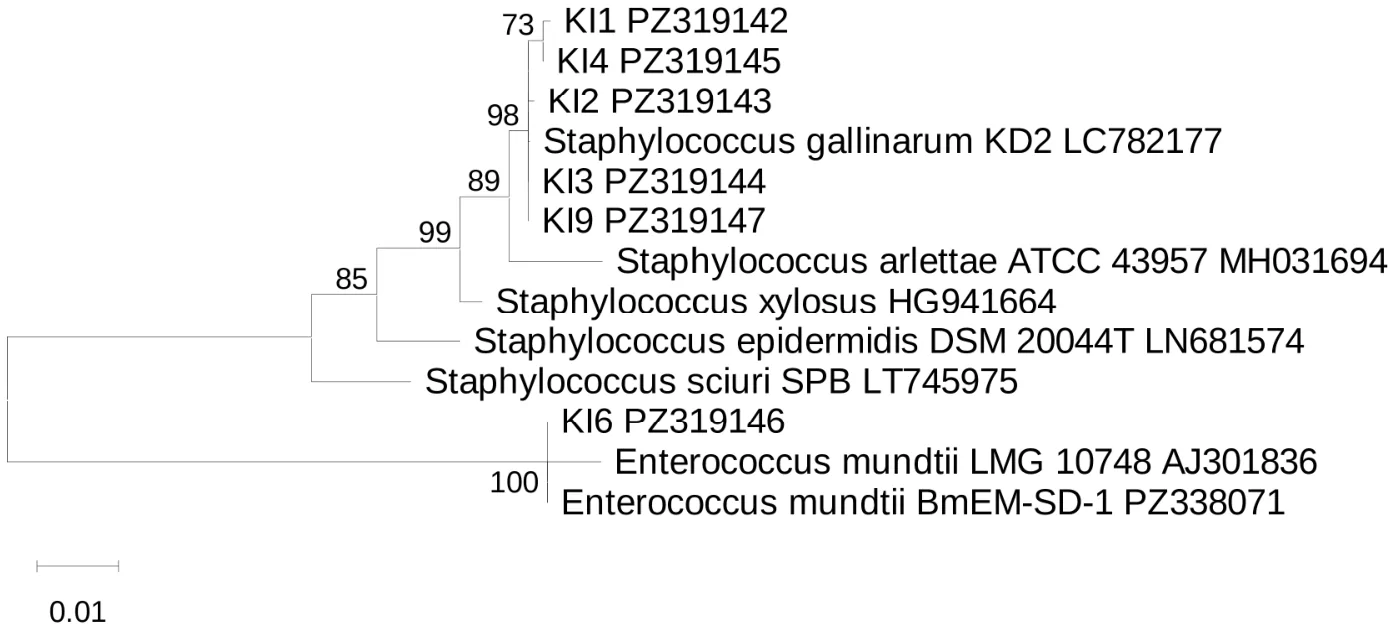
Maximum-likelihood phylogenetic tree based on partial 16S rRNA gene sequences of six gut-associated bacterial isolates from *Bombyx mori* and seven selected reference sequences. The phylogeny was inferred in MEGA12 using the Maximum Likelihood method and the Tamura–Nei nucleotide substitution model. The tree with the highest log likelihood (−3863.96) is shown. Bootstrap support percentages from 1000 replicates are shown next to the branches. Branch lengths represent substitutions per site; the scale bar indicates 0.01 substitutions per site. Isolate sequences generated in this study are labeled by KI designation and GenBank accession number. The complete multiple sequence alignment corresponding to Figure 2, containing 13 nucleotide sequences and 1871 aligned positions, is provided as Supplementary File S3.

BLAST analysis of the deposited consensus sequences showed 100% query coverage and 99.93–100% sequence identity for all six isolates (Table 1). The top BLAST hits for the staphylococcal isolates included sequences annotated as *Staphylococcus gallinarum*, *Staphylococcus arlettae*, or *Staphylococcus* sp., whereas KI6 showed 100% identity to *Enterococcus mundtii*. In the maximum-likelihood phylogeny, isolates KI1, KI2, KI3, KI4, and KI9 clustered with the *S. gallinarum* reference sequence, while KI6 grouped with *E. mundtii* reference sequences (Figure 2). Based on the combined BLAST and phylogenetic evidence, KI1, KI2, KI3, KI4, and KI9 were assigned to *S. gallinarum*, whereas KI6 was assigned to *E. mundtii*.

### 3.3. Antibiotic Disk-Diffusion Profiles of Selected Bacterial Isolates

Antibiotic-response profiles of the selected isolates were characterized descriptively from inhibition-zone diameters (Table 2). Detectable zones were observed for all six isolates with chloramphenicol, penicillin, tetracycline, gentamicin, and amoxicillin. Streptomycin produced a 9.0-mm zone only for KI6, whereas no detectable zone was observed for the other isolates. For erythromycin, measurable zones were observed for KI2, KI4, KI6, and KI9, whereas no detectable zone was observed for KI1 or KI3. Because taxon-specific validated breakpoints were not applied, these measurements are not interpreted as clinical susceptibility categories.

### 3.4. Solvent Affinity of Selected Bacterial Isolates

Solvent-dependent partitioning was measured for KI2, KI4, KI6, and KI9 and varied among these isolates and between solvents (Table 3). Affinity values with ethyl acetate ranged from 7.33% to 20.18%, whereas values with chloroform ranged from 32.63% to 38.57%. These measurements are reported as comparative solvent-affinity values and are not interpreted as evidence of intestinal adhesion or colonization.

### 3.5. Antagonistic Screening and Selection of Staphylococcus gallinarum KI4

Among the four isolates evaluated in the antagonistic screening (KI2, KI4, KI6, and KI9), *S. gallinarum* KI2 and KI4 produced detectable inhibition zones against all three indicator bacteria, whereas *S. gallinarum* KI9 and *E. mundtii* KI6 produced no detectable inhibition zones under the assay conditions. KI2 and KI4 were therefore retained for quantitative comparison. Their total inhibition-zone diameters, including the 6-mm well, ranged from 14.0 to 18.0 mm (Table 4). KI4 was subsequently selected for in vivo evaluation because it produced the largest measured zone against *B. cereus*. These in vitro assays used whole bacterial cultures and therefore do not identify the inhibitory factor or establish that the same process accounts for the in vivo host response.

### 3.6. Effects of Staphylococcus gallinarum KI4 Under Bacillus cereus Challenge

#### 3.6.1. Effects of *Staphylococcus gallinarum* KI4 on Growth Performance and Cocoon Traits

Growth performance and cocoon-related traits were evaluated under non-challenged conditions and *B. cereus* challenge (Table 5). Under non-challenged conditions, G2 did not differ significantly from G1 in final larval weight, cocooning, pupation, fresh cocoon weight, or shell weight (Tukey HSD, *p* > 0.05 for G1–G2 comparisons). Thus, the KI4-only treatment was not associated with a statistically significant increase in shell weight in the corrected analysis.

*B. cereus* challenge alone (G3) was associated with significantly lower final larval weight, cocooning, pupation, fresh cocoon weight, and shell weight than the untreated control (G1) (*p* < 0.05). Compared with G3, larvae receiving KI4 supplementation initiated before challenge and continued once daily thereafter (G4) had significantly higher final larval weight, cocooning, pupation, fresh cocoon weight, and shell weight (*p* < 0.05). Fresh cocoon weight in G4 was also significantly higher than in G1 and G2. These results describe treatment-associated differences under the tested regimen and do not identify the mechanism responsible. All five Table 5 omnibus ANOVA tests remained significant after Holm–Bonferroni adjustment (largest adjusted *p* = 0.00236 for shell weight; see Supplementary File S2, Holm–Bonferroni worksheet).

#### 3.6.2. Effect of *Staphylococcus gallinarum* KI4 on Endpoint Survival Under *Bacillus cereus* Challenge

**Figure 3:**
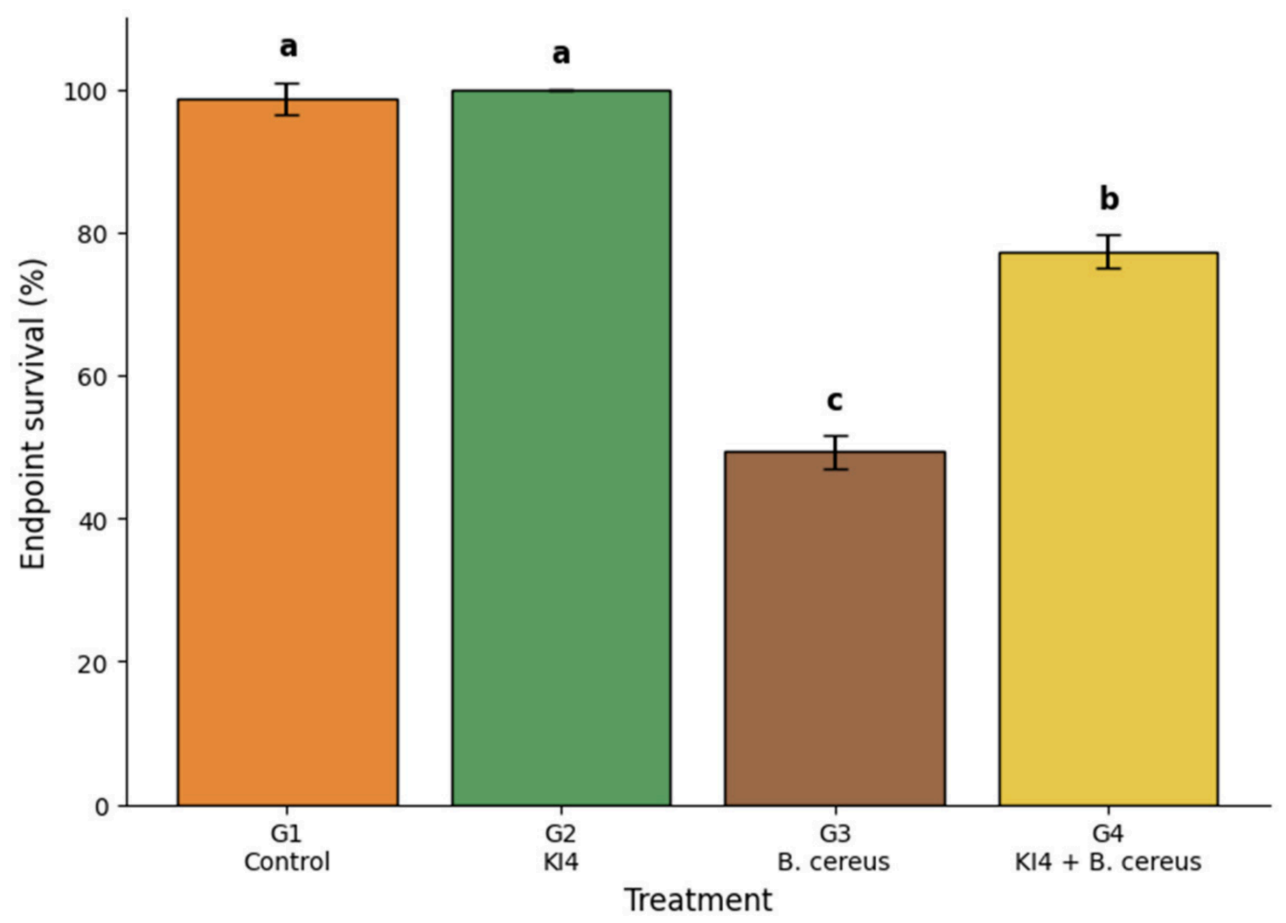
Endpoint survival of *Bombyx mori* under untreated, KI4, *Bacillus cereus*, and KI4 + *Bacillus cereus* treatments. Bars represent mean ± SD from three independent rearing replicates (*n* = 3), with 25 larvae initially included in each replicate. Different lowercase letters indicate significant differences among treatments according to one-way ANOVA followed by Tukey’s HSD test (*p* < 0.05).

Endpoint survival differed significantly among treatment groups (Figure 3). Larvae challenged with *B. cereus* alone (G3) had lower endpoint survival than the untreated control (G1), with replicate-level survival of 48%, 52%, and 48% (mean ± SD, 49.33 ± 2.31%). Larvae receiving KI4 alone (G2) had 100% endpoint survival in all three replicates and did not differ significantly from G1.

Larvae receiving daily KI4 supplementation initiated before *B. cereus* challenge and continued thereafter (G4) had significantly higher endpoint survival than the pathogen-only group G3 (77.33 ± 2.31% vs. 49.33 ± 2.31%; Tukey-adjusted *p* < 0.001). Under the tested regimen, this result supports an association between KI4 supplementation and higher endpoint survival but does not establish that the response was mediated by the antagonistic activity observed in vitro. Replicate-level survival data supporting Figure 3 are provided in Supplementary Table S1.

## 4. Discussion

This study combined culture-based screening, molecular identification, in vitro antagonistic screening, and a host challenge experiment to evaluate a gut-associated bacterial isolate as a potential resource for silkworm production. Two principal observations were obtained. First, *S. gallinarum* KI4 showed detectable in vitro inhibition against the three tested bacterial indicators. Second, KI4 supplementation was associated with higher endpoint survival and developmental outcomes during *B. cereus* challenge under the tested feeding regimen. These observations should be considered separately because the present study did not quantify *B. cereus* burden, KI4 persistence, or changes in the endogenous gut bacterial community and did not include a non-antagonistic bacterial supplementation control. The study therefore does not establish that the in vivo response was mediated by the antagonistic activity observed in vitro. Comparable benefits of host-associated or probiotic bacteria on growth, production traits, stress tolerance, or pathogen resistance have been reported in insects and silkworms [9,10,11,12,13,14,15], although defensive microbial associations can involve diverse and context-dependent mechanisms [42]. Accordingly, the in vitro inhibition should not be interpreted as evidence that KI4 suppressed *B. cereus* within the silkworm gut or altered the resident gut bacterial community in vivo. The contribution of the present study is therefore framed around isolate-level screening and the host-level challenge outcome rather than mechanistic resolution.

The isolation procedure was intentionally selective. Enrichment in MRS medium at pH 10 provided a strongly alkaline selective condition relevant to the larval gut environment and recovered only the subset of bacteria able to grow under those conditions. Thus, this culture-based approach was selected to identify bacteria that could be propagated reproducibly for subsequent screening, not to represent the complete gut community. Insect gut microbiota is environmentally responsive and structured by multiple ecological interactions [1,2,3,4,5,6,7,8]. In *B. mori*, a recent longitudinal analysis shows that feed type and developmental time can substantially alter intestinal bacterial community structure [8]. In addition, the 50 colonies originated from one pooled sample of 20 midguts. The isolates therefore cannot be interpreted as a comprehensive representation of the Nang Lai gut microbiota, and the study does not demonstrate that KI4 is dominant or consistently present across individual larvae. Independent midgut pools and strain-specific detection would be necessary to establish prevalence.

The isolation method combined alkaline enrichment with subsequent anaerobic incubation of MRS-CaCO_3_ agar plates. Recovery of KI4 under these culture conditions indicates that it could form colonies during anaerobic cultivation. However, isolation under anaerobic conditions alone does not establish the isolate’s oxygen requirements or comparative growth performance in the presence and absence of oxygen.

Molecular identification was strengthened by combining bidirectional 16S rRNA sequencing, BLAST comparison, and maximum-likelihood phylogenetic analysis. The top BLAST annotations among the staphylococcal isolates included *Staphylococcus gallinarum*, *Staphylococcus arlettae*, and *Staphylococcus* sp., whereas KI6 matched *Enterococcus mundtii*. In the updated phylogeny, KI1, KI2, KI3, KI4, and KI9 clustered with the *S. gallinarum* reference sequence, whereas KI6 grouped with *E. mundtii* reference sequences. Based on the combined BLAST and phylogenetic evidence, the isolates are assigned to these species in the present manuscript. Nevertheless, partial 16S rRNA sequences can have limited discriminatory power among closely related staphylococci; whole-genome sequencing or additional discriminatory loci would provide higher-resolution taxonomic confirmation and would also facilitate screening for virulence factors and transferable antimicrobial-resistance determinants.

The antibiotic disk-diffusion and solvent-partitioning assays were retained as comparative isolate characterizations but were interpreted conservatively. Antibiotic-response profiles are reported as measured inhibition-zone diameters because taxon-specific validated clinical breakpoints and full quality-control validation were not available for all isolates under the study conditions [34,43,44,45]. Likewise, solvent-dependent partitioning reflects physicochemical surface behavior under in vitro conditions and does not demonstrate epithelial adhesion or gut colonization [35,36,37,38,39,40]. Recent work with dietary *B. subtilis* in silkworms reported improved growth and cocoon traits despite no detectable midgut colonization, reinforcing that host benefit and intestinal persistence are distinct endpoints [14]. Claims of effective colonization and stable integration into the resident microbiota are therefore avoided. Recovery of KI4 from the gut by selective culture, strain-specific PCR, quantitative PCR, or labeling would be required to establish persistence.

KI2 and KI4 produced inhibition zones against *B. cereus*, *B. thuringiensis*, and *S. marcescens*, whereas KI9 and KI6 did not, indicating that antagonism was isolate-specific rather than a uniform species trait. The total diameters included the 6-mm well, so the net inhibitory margin surrounding the well was modest. The results therefore support detectable antagonism, but not exceptionally strong antimicrobial activity. Because whole cultures were used, the assay cannot distinguish acidification, nutrient competition, viable-cell effects, hydrogen peroxide or other oxidative metabolites, or secreted antimicrobial compounds [6,17,18,19,42]. Tests using cell-free supernatant, neutralized supernatant, heat treatment, protease treatment, and hydrogen-peroxide controls would help resolve the basis of inhibition.

KI4 showed detectable in vitro inhibition of the tested bacterial indicators, but inhibitory activity was not compared under different oxygen conditions. Therefore, the present results do not show whether its pathogen-inhibitory activity differs between aerobic and anaerobic environments. Future work should compare KI4 growth and antagonistic activity under controlled oxygen conditions to determine whether oxygen availability influences the observed inhibition.

The in vivo experiment showed that daily KI4 supplementation initiated before *B. cereus* challenge and continued thereafter was associated with higher endpoint survival (77.33 ± 2.31% in G4 versus 49.33 ± 2.31% in G3), as well as higher cocooning and pupation. The endpoint was assessed at the end of the fifth instar immediately before mounting; individual time-to-event data were not analyzed, so no Kaplan–Meier curve or hazard estimate is inferred. The design also lacked a non-antagonistic bacterial control such as KI9 or KI6 and did not include fully matched PBS-treated and fasting controls across all groups. Consequently, the in vivo response cannot be attributed specifically to KI4 antagonism or distinguished completely from other effects of bacterial supplementation, vehicle handling, or the treatment regimen. Future experiments should include a non-antagonistic isolate, matched vehicle and fasting controls, dose–response treatments, quantification of pathogen burden, and recovery of KI4 from the intestine. In the corrected Table 5 analysis, KI4 alone did not significantly increase shell weight relative to the untreated control. Therefore, no conclusion concerning enhanced shell deposition, silk-production efficiency, equivalence, or safety is drawn from the non-challenged comparison. A nonspecific effect of bacterial supplementation therefore cannot be excluded. Targeted microbiome analysis would also be required to determine whether KI4 administration altered the resident bacterial community during challenge.

From the perspective of resource-insect utilization, the study identifies a host-derived bacterial candidate that may be incorporated into future disease-management strategies for silkworm production. Until identity, safety, efficacy, and host benefit are demonstrated under the intended conditions, KI4 should be described as a candidate biological resource rather than as a probiotic product [20,21]. Before practical use, several requirements remain: definitive taxonomic confirmation, assessment of hemolysis and virulence-associated genes, characterization of antimicrobial-resistance determinants [43,44,45], demonstration of gut persistence, identification of the active inhibitory mechanism, formulation and storage studies, and validation under farm-rearing conditions. Protective microbial associations may provide useful conceptual guidance, but practical development requires evidence specific to the candidate strain and production system [42]. Addressing these points would determine whether KI4 can progress from a laboratory candidate to a safe and reproducible sericulture input.

A possible future application pathway is delivery of KI4 on mulberry leaves, consistent with the feeding approach used in this study. Further work would need to optimize dosing and formulation, assess cell viability and stability on treated leaves, and validate reproducible host benefits under different rearing conditions. Importantly, strain-level biosafety evaluation and field efficacy studies must precede any recommendation for practical sericulture use.

## 5. Conclusions

Culture-based screening of gut-associated bacteria from the Nang Lai silkworm strain identified KI4, assigned to *Staphylococcus gallinarum* based on partial 16S rRNA gene sequence analysis and phylogenetic comparison, as an isolate with detectable in vitro antagonistic activity. In the in vivo experiment, KI4 supplementation was associated with higher endpoint survival, cocooning, and pupation during *B. cereus* challenge under the tested feeding regimen. The in vitro antagonistic activity and the in vivo host response represent separate experimental observations, and the present study does not establish a causal mechanistic link between them. Because a non-antagonistic bacterial supplementation control was not included, the observed in vivo response cannot be attributed specifically to the antagonistic activity of KI4. The corrected statistical analysis did not show a significant increase in cocoon shell weight in the KI4-only group relative to the untreated control. The study also does not demonstrate intestinal persistence or colonization of KI4, alteration of the endogenous gut microbiota, a specific inhibitory mechanism, equivalence or comprehensive safety, or field efficacy. These findings support further controlled evaluation of *S. gallinarum* KI4 as a candidate host-derived bacterial resource for sericulture. Strain-level biosafety assessment and validation under practical rearing conditions are required before KI4 can be considered for application in sericulture. Given the small number of independent rearing replicates and multiple outcomes assessed, these treatment-associated findings should be considered exploratory pending independent confirmation.

## Figures and Tables

**Table 1:** BLAST-based sequence characteristics and phylogenetic assignments of representative gut-associated bacterial isolates based on partial 16S rRNA gene sequences.

Isolate (Accession)	Length (bp)	Coverage (%)	Identity (%)	Top BLAST Hit	Phylogenetic Assignment
KI1 (PZ319142)	1416	100	99.93	PQ045247 *Staphylococcus* sp. strain TAST11	*Staphylococcus gallinarum*
KI2 (PZ319143)	1406	100	99.93	PP514634 *Staphylococcus arlettae* strain XQYXY-5	*Staphylococcus gallinarum*
KI3 (PZ319144)	1416	100	100	PP514634 *Staphylococcus arlettae* strain XQYXY-5	*Staphylococcus gallinarum*
KI4 (PZ319145)	1406	100	100	PP486276 *Staphylococcus* sp. strain L-24	*Staphylococcus gallinarum*
KI6 (PZ319146)	1261	100	100	PZ338071 *Enterococcus mundtii* strain BmEM-SD-1	*Enterococcus mundtii*
KI9 (PZ319147)	1403	100	100	LC782173 *Staphylococcus gallinarum* KB2	*Staphylococcus gallinarum*

Note: The top BLAST hit is the database match reported as the top hit in the updated BLAST analysis. Phylogenetic assignment reflects the combined BLAST and maximum-likelihood phylogenetic evidence rather than the top BLAST annotation alone.

**Table 2:** Antibiotic disk-diffusion inhibition-zone diameters (mm) of selected bacterial isolates.

Isolate	Species (GenBank Accession)	C30	P10	TE30	GEN10	AMS30	S10	E15
KI1	*Staphylococcus gallinarum (PZ319142)*	25.4	17.5	30.0	24.8	29.0	ND	ND
KI2	*Staphylococcus gallinarum (PZ319143)*	25.6	15.8	30.0	24.8	29.3	ND	14.0
KI3	*Staphylococcus gallinarum (PZ319144)*	25.0	15.5	29.8	24.5	29.0	ND	ND
KI4	*Staphylococcus gallinarum (PZ319145)*	25.8	19.8	30.5	24.3	29.3	ND	15.5
KI6	*Enterococcus mundtii (PZ319146)*	25.2	17.8	30.0	19.3	30.0	9.0	22.0
KI9	*Staphylococcus gallinarum (PZ319147)*	24.7	15.0	30.0	22.8	28.8	ND	15.5

C30, chloramphenicol 30 µg; P10, penicillin 10 units; TE30, tetracycline 30 µg; GEN10, gentamicin 10 µg; AMS30, amoxicillin 30 µg; S10, streptomycin 10 µg; E15, erythromycin 15 µg. ND, no detectable inhibition zone. Values are measured inhibition-zone diameters (mm) and are reported descriptively; no clinical susceptibility categories were assigned.

**Table 3:** Solvent affinity (%) of selected bacterial isolates measured by partitioning with ethyl acetate and chloroform.

Isolate	Species	Solvent Affinity (%)	
		Ethyl Acetate (%)	Chloroform (%)
KI2	*Staphylococcus gallinarum* (PZ319143)	20.18 ± 10.77	37.00 ± 14.81
KI4	*Staphylococcus gallinarum* (PZ319145)	11.80 ± 6.91	38.57 ± 12.58
KI6	*Enterococcus mundtii* (PZ319146)	10.40 ± 3.76	34.85 ± 3.30
KI9	*Staphylococcus gallinarum* (PZ319147)	7.33 ± 2.87	32.63 ± 10.77

Values are presented as mean ± SD from three assay replicates (*n* = 3).

**Table 4:** Quantitative inhibition-zone diameters (mm) produced by selected bacterial isolates against bacterial pathogens.

Isolate	Species	*Serratia marcescens*	*Bacillus cereus*	*Bacillus thuringiensis*
KI2	*Staphylococcus gallinarum*	15.0 ± 0.8	17.0 ± 0.5	15.0 ± 0.6
KI4	*Staphylococcus gallinarum*	14.0 ± 0.6	18.0 ± 1.1	15.0 ± 0.8

Values are presented as mean ± SD from three replicate assay plates (*n* = 3). Total inhibition-zone diameters include the 6-mm well. KI9 and KI6 produced no detectable inhibition zones under the assay conditions and were therefore not included in the quantitative comparison. Sterile MRS broth was used as the negative control. A separate inhibition-zone result for the sterile-MRS negative control is not available in the archived laboratory record and is therefore not reported.

**Table 5:** Growth performance and cocoon traits of *Bombyx mori* under different treatments.

Treatment	Growth Performance			Cocoon Traits	
	Final Larval Weight (g/Larva)	Cocooning (%)	Pupation (%)	Fresh Cocoon Weight (g)	Shell Weight (g)
G1—Control	2.10 ± 0.01 ^a^	98.67 ± 2.31 ^a^	96.00 ± 4.00 ^a^	1.14 ± 0.01 ^b^	0.15 ± 0.01 ^a^
G2—*Staphylococcus gallinarum* KI4	2.12 ± 0.01 ^a^	100.00 ± 0.00 ^a^	100.00 ± 0.00 ^a^	1.15 ± 0.01 ^b^	0.17 ± 0.00 ^a^
G3—*Bacillus cereus*	1.99 ± 0.02 ^b^	49.33 ± 2.31 ^c^	48.00 ± 0.00 ^c^	0.99 ± 0.02 ^c^	0.09 ± 0.04 ^b^
G4—*Staphylococcus gallinarum* KI4 + *Bacillus cereus*	2.10 ± 0.01 ^a^	77.33 ± 2.31 ^b^	77.33 ± 2.31 ^b^	1.20 ± 0.00 ^a^	0.15 ± 0.01 ^a^

Values are presented as mean ± SD of three independent rearing replicates (*n* = 3), with 25 larvae initially included in each replicate. Different lowercase letters within the same column indicate significant differences among treatments according to one-way ANOVA followed by Tukey’s HSD test (*p* < 0.05). Given the limited number of rearing replicates and the difficulty of verifying ANOVA assumptions, all inferential comparisons, including shell-weight contrasts, should be interpreted as exploratory.

## Data Availability

The 16S rRNA gene sequences generated in this study are available in GenBank under accession numbers PZ319142-PZ319147. Replicate-level data underlying Table 5 and Figure 3 are provided in Supplementary Table S1, and the corresponding statistical output is provided in Supplementary File S2. Additional raw data supporting the conclusions of this article will be made available by the corresponding author on request. Supplementary Table S1 also reports the corresponding numbers of surviving, cocooning, and pupating larvae using the initial denominator of 25 larvae per replicate. The multiple sequence alignment corresponding to Figure 2 is provided as Supplementary File S3.

## Supplementary Materials

The following supporting information can be downloaded at: https://www.mdpi.com/article/10.3390/insects17101075/s1. Supplementary Figure S1: Original full and uncropped agarose gel image corresponding to Figure 1C (M, DNA size marker); Supplementary Table S1: Replicate-level data for growth performance, cocoon traits, and endpoint survival; Supplementary File S2: Statistical analysis output for growth performance, cocoon traits, and endpoint survival; Supplementary File S3: Multiple sequence alignment corresponding to Figure 2.

## References

[1] Engel P., Moran N.A. (2013). The gut microbiota of insects—Diversity in structure and function. FEMS Microbiol. Rev..

[2] Douglas A.E. (2015). Multiorganismal insects: Diversity and function of resident microorganisms. Annu. Rev. Entomol..

[3] Zhang X., Zhang F., Lu X. (2022). Diversity and functional roles of the gut microbiota in lepidopteran insects. Microorganisms.

[4] Wang Y., Tang J., Chen Y., Chen S., Chen S., Yu X., Wan C., Xiang G., Chen Y., Li Q. (2025). The ecological–evolutionary game of the insect gut microbiome: Environmental drivers, host regulation, and prospects for cross-cutting applications. Vet. Sci..

[5] Muhammad A., Sun C., Shao Y. (2026). Ecological and functional dynamics of gut microbiota in the model insect, silkworm *Bombyx mori*. World J. Microbiol. Biotechnol..

[6] Hibbing M.E., Fuqua C., Parsek M.R., Peterson S.B. (2010). Bacterial competition: Surviving and thriving in the microbial jungle. Nat. Rev. Microbiol..

[7] Coyte K.Z., Schluter J., Foster K.R. (2015). The ecology of the microbiome: Networks, competition, and stability. Science.

[8] Chen C., Li M., Li F., Liang X., Zhang H., Gu Y., Guo G. (2024). Dynamic alterations of the intestinal microbiota of fifth-instar silkworms (*Bombyx mori*) fed an artificial diet or mulberry leaves. Insects.

[9] Pradeep S., Johnson Thangaraj Edward Y.S., Angappan S., Murugaiyan S., Ramasamy S.V., Boopathi N.M. (2025). Lactic acid bacteria: A probiotic to mitigate pesticide stress in honey bee. Probiotics Antimicrob. Proteins.

[10] Grau T., Vilcinskas A., Joop G. (2017). Probiotic *Enterococcus mundtii* isolate protects the model insect *Tribolium castaneum* against *Bacillus thuringiensis*. Front. Microbiol..

[11] Lecocq A., Natsopoulou M.E., Berggreen I., Eilenberg J., Heckmann L.-H.L., Nielsen H.V., Stensvold C.R., Jensen A.B. (2021). Probiotic properties of an indigenous *Pediococcus pentosaceus* strain on *Tenebrio molitor* larval growth and survival. J. Insects Food Feed..

[12] Suraporn S., Liu J., Ren F., Wang L., Feng M., Terenius O., Swevers L. (2025). Towards a rational basis for the selection of probiotics to improve silkworm health and performance. Insects.

[13] Zeng Z., Tong X., Yang Y., Zhang Y., Deng S., Zhang G., Dai F. (2024). *Pediococcus pentosaceus* ZZ61 enhances growth performance and pathogenic resistance of silkworm *Bombyx mori* by regulating gut microbiota and metabolites. Bioresour. Technol..

[14] Ren C., Meng Y., Liu Y., Wang Y., Wang H., Liu Y., Liu C., Fan X., Zhang S. (2025). Probiotic *Bacillus subtilis* enhances silkworm (*Bombyx mori*) growth performance and silk production via modulating gut microbiota and amino acid metabolism. Anim. Microbiome.

[15] Zou Y., Ren Y., Qu Y., Xiao Y., Xing D., Zhang W., Zhou D., Liu J., Li Q. (2026). Effects of *Lactobacillus* supplementation on gut microbiota, short-chain fatty acids, and nutrient metabolism in silkworm (*Bombyx mori*). J. Insect Physiol..

[16] Charteris W.P., Kelly P.M., Morelli L., Collins J.K. (1998). Development and application of an in vitro methodology to determine the transit tolerance of potentially probiotic *Lactobacillus* and *Bifidobacterium* species in the upper human gastrointestinal tract. J. Appl. Microbiol..

[17] Bermudez-Brito M., Plaza-Díaz J., Muñoz-Quezada S., Gómez-Llorente C., Gil A. (2012). Probiotic mechanisms of action. Ann. Nutr. Metab..

[18] Oelschlaeger T.A. (2010). Mechanisms of probiotic actions—A review. Int. J. Med. Microbiol..

[19] Imlay J.A. (2013). The molecular mechanisms and physiological consequences of oxidative stress in bacteria. Nat. Rev. Microbiol..

[20] Food and Agriculture Organization of the United Nations (FAO), World Health Organization (WHO) (2002). Guidelines for the Evaluation of Probiotics in Food.

[21] Hill C., Guarner F., Reid G., Gibson G.R., Merenstein D.J., Pot B., Morelli L., Canani R.B., Flint H.J., Salminen S. (2014). The International Scientific Association for Probiotics and Prebiotics consensus statement on the scope and appropriate use of the term probiotic. Nat. Rev. Gastroenterol. Hepatol..

[22] Altman G.H., Diaz F., Jakuba C., Calabro T., Horan R.L., Chen J., Lu H., Richmond J., Kaplan D.L. (2003). Silk-based biomaterials. Biomaterials.

[23] Meng X., Zhu F., Chen K. (2017). Silkworm: A promising model organism in life science. J. Insect Sci..

[24] Awais M.M., Fei S., Xia J., Feng M., Sun J. (2024). Insights into midgut cell types and their crucial role in antiviral immunity in the lepidopteran model *Bombyx mori*. Front. Immunol..

[25] Li G.N., Xia X.J., Zhao H.H., Sendegeya P., Zhu Y. (2015). Identification and characterization of *Bacillus cereus* SW7-1 in *Bombyx mori* (Lepidoptera: Bombycidae). J. Insect Sci..

[26] Qadir J., Bhat S.A., Rasheed S. (2025). Bacterial pathogens of silkworm: Impacts and management strategies. IJBSM.

[27] Raymond B., Johnston P.R., Nielsen-LeRoux C., Lereclus D., Crickmore N. (2010). *Bacillus thuringiensis*: An impotent pathogen?. Trends Microbiol..

[28] Zhang Y., Shang R., Zhang J., Li J., Zhu G., Yao M., Sun J., Shen Z. (2020). Isolation and identification of two *Serratia marcescens* strains from silkworm, *Bombyx mori*. Antonie Van Leeuwenhoek.

[29] Nazar M.Z., Umer A., Sarwar Z.M., Faiz F.U., Hussain S. (2020). Haemolymph assay and morphological changes of silkworm, *Bombyx mori* L. (Bombycidae: Lepidoptera) infected with *Bacillus thuringiensis* var. sotto. Pak. J. Agric. Res..

[30] Kumar S., Stecher G., Suleski M., Sanderford M., Sharma S., Tamura K. (2024). MEGA12: Molecular Evolutionary Genetic Analysis Version 12 for Adaptive and Green Computing. Mol. Biol. Evol..

[31] Tamura K., Nei M. (1993). Estimation of the number of nucleotide substitutions in the control region of mitochondrial DNA in humans and chimpanzees. Mol. Biol. Evol..

[32] Felsenstein J. (1985). Confidence limits on phylogenies: An approach using the bootstrap. Evolution.

[33] Saitou N., Nei M. (1987). The neighbor-joining method: A new method for reconstructing phylogenetic trees. Mol. Biol. Evol..

[34] Bauer A.W., Kirby W.M.M., Sherris J.C., Turck M. (1966). Antibiotic susceptibility testing by a standardized single disk method. Am. J. Clin. Pathol..

[35] Rosenberg M., Gutnick D., Rosenberg E. (1980). Adherence of bacteria to hydrocarbons: A simple method for measuring cell-surface hydrophobicity. FEMS Microbiol. Lett..

[36] Krausova G., Hyrslova I., Hynstova I. (2019). *In Vitro* Evaluation of Adhesion Capacity, Hydrophobicity, and Auto-Aggregation of Newly Isolated Potential Probiotic Strains. Fermentation.

[37] van Oss C.J. (2003). Long-range and short-range mechanisms of hydrophobic attraction and hydrophilic repulsion in specific and aspecific interactions. J. Mol. Recognit..

[38] Del Re B., Sgorbati B., Miglioli M., Palenzona D. (2000). Adhesion, autoaggregation and hydrophobicity of 13 strains of *Bifidobacterium longum*. Lett. Appl. Microbiol..

[39] Rosenberg M. (2006). Microbial adhesion to hydrocarbons: Twenty-five years of doing MATH. FEMS Microbiol. Lett..

[40] Monteagudo-Mera A., Rastall R.A., Gibson G.R., Charalampopoulos D., Chatzifragkou A. (2019). Adhesion mechanisms mediated by probiotics and prebiotics and their potential impact on human health. Appl. Microbiol. Biotechnol..

[41] Balouiri M., Sadiki M., Ibnsouda S.K. (2016). Methods for *in vitro* evaluating antimicrobial activity: A review. J. Pharm. Anal..

[42] Flórez L.V., Biedermann P.H.W., Engl T., Kaltenpoth M. (2015). Defensive symbioses of animals with prokaryotic and eukaryotic microorganisms. Nat. Prod. Rep..

[43] Mathur S., Singh R. (2005). Antibiotic resistance in food lactic acid bacteria—A review. Int. J. Food Microbiol..

[44] Gueimonde M., Sánchez B., de Los Reyes-Gavilán C.G., Margolles A. (2013). Antibiotic resistance in probiotic bacteria. Front. Microbiol..

[45] Allen H.K., Donato J., Wang H.H., Cloud-Hansen K.A., Davies J., Handelsman J. (2010). Call of the wild: Antibiotic resistance genes in natural environments. Nat. Rev. Microbiol..

