# Supplementary material for: Isolation and Evaluation of the Gut-Associated Bacterium Staphylococcus gallinarum KI4: Effects on Survival and Cocoon Traits of Bombyx mori Under Bacillus cereus Challenge
Source: Insects. 2026 Oct 17;17(10):1075. doi: 10.3390/insects17101075 (PMC13705185; doi:10.3390/insects17101075)
Supplement: Supplementary file 1 [file insects-17-01075-s001.zip › Supplementary_File_S3_Full_Alignment.pdf]

Multiple sequence alignment corresponding to the phylogenetic tree in Figure 2.

```
KI1_PZ319142                                      --------------------------------------------------TGCAGTCGAG
KI2_PZ319143                                      ---------------------------------------------------CAAGTCGAG
KI3_PZ319144                                      ---------------------------------------------------CAAGTCGAG
KI4_PZ319145                                      ---------------------------------------------------GCAGTCGAG
KI6_PZ319146                                      ------------------------------------------------------------
KI9_PZ319147                                      ------------------------------------------------------GTCGAG
Staphylococcus_sciuri_SPB_LT745975                ---------------------------------------------------TGCGTCGAG
Staphylococcus_xylosus_HG941664                   TAGAGTTTGATCCTGGCTCAGGATGAACGCTGGCGGCGTGCCTAATACATGCAAGTCGAG
Staphylococcus_epidermidis_DSM_20044T_LN681574    ---------------------GATGAACGCTGGCGGCGTGCCTAATACATGCAAGTCGAG
Enterococcus_mundtii_LMG_10748_AJ301836           -AGAGTTTGATNNTGGCTCAGGACGAACGCTGGCGGCGTGCCTAATACATGCAAGTCGAA
Staphylococcus_gallinarum_KD2_LC782177            -------------------------------------------------TGCAAGTCGAG
Enterococcus_mundtii_BmEM-SD-1_PZ338071           -------------------------------------------------TGCAAGTCGAA
Staphylococcus_arlettae_ATCC_43957_MH031694       --------------------------------GCGGCGTGCTTAATACATGCAAGTTGAG

KI1_PZ319142                                      CGAACAGATAA-----GGAGCTTGCTCCTTTG-----ACGTTAGCGGCGGACGGGTGAGT
KI2_PZ319143                                      CGAACAGATAA-----GGAGCTTGCTCCTTTG-----ACGTTAGCGGCGGACGGGTGAGT
KI3_PZ319144                                      CGAACAGATAA-----GGAGCTTGCTCCTTTG-----ACGTTAGCGGCGGACGGGTGAGT
KI4_PZ319145                                      CGAACAGATAA-----GGAGCTTGCTCCTTTG-----ACGTTAGCGGCGGACGGGTGAGT
KI6_PZ319146                                      ------------------------------------------------------------
KI9_PZ319147                                      CGAACAGATAA-----GGAGCTTGCTCCTTTG-----ACGTTAGCGGCGGACGGGTGAGT
```

```
Staphylococcus_sciuri_SPB_LT745975                CGAACAGATGA-----GAAGCTTGCTTCTCTG-----ATGTTAGCGGCGGACGGGTGAGT
Staphylococcus_xylosus_HG941664                   CGAACAGATAA-----GGAGCTTGCTCCTTTG-----ACGTTAGCGGCGGACGGGTGAGT
Staphylococcus_epidermidis_DSM_20044T_LN681574    CGAACAGACGA-----GGAGCTTGCTCCTCTG-----ACGTTAGCGGCGGACGGGTGAGT
Enterococcus_mundtii_LMG_10748_AJ301836           CGCTTCTTTTCCCACCG-AGCTTGCTCCACCGGGAAAAGAAGAGTGACGAACGGGTGAGT
Staphylococcus_gallinarum_KD2_LC782177            CGAACAGATAA-----GGAGCTTGCTCCTTTG-----ACGTTAGCGGCGGACGGGTGAGT
Enterococcus_mundtii_BmEM-SD-1_PZ338071           CGCTTCTTTTCCCACCGGAGCTTGCTCCACCGGGAAAAGAGGAGTGGCGAACGGGTGAGT
Staphylococcus_arlettae_ATCC_43957_MH031694       CGAACAGATAA-----GGAGCTTGCTCCTTCG-----ACGTTAGCGGCGGACGGGTGAGT

KI1_PZ319142                                      AACACGTGGGTAACCTACCTATAAGACTGGAATAACTCCGGGAAACCGGGGCTAA-TGCC
KI2_PZ319143                                      AACACGTGGGTAACCTACCTATAAGACTGGAATAACTCCGGGAAACCGGGGCTAA-TGCC
KI3_PZ319144                                      AACACGTGGGTAACCTACCTATAAGACTGGAATAACTCCGGGAAACCGGGGCTAA-TGCC
KI4_PZ319145                                      AACACGTGGGTAACCTACCTATAAGACTGGAATAACTCCGGGAAACCGGGGCTAA-TGCC
KI6_PZ319146                                      ------------------------------------------------------------
KI9_PZ319147                                      AACACGTGGGTAACCTACCTATAAGACTGGAATAACTCCGGGAAACCGGGGCTAA-TGCC
Staphylococcus_sciuri_SPB_LT745975                AACACGTGGGTAACCTACCTATAAGACTGGGATAACTCCGGGAAACCGGGGCTAA-TACC
Staphylococcus_xylosus_HG941664                   AACACGTGGGTAACCTACCTATAAGACTGGGATAACTTCGGGAAACCGGAGCTAA-TACC
Staphylococcus_epidermidis_DSM_20044T_LN681574    AACACGTGGATAACCTACCTATAAGACTGGGATAACTTCGGGAAACCGGAGCTAA-TACC
Enterococcus_mundtii_LMG_10748_AJ301836           AACACGTGGGTAACCTGCCCATCAGAAGGGTATAACACTTGGAAACAGGTGCTAAATACC
Staphylococcus_gallinarum_KD2_LC782177            AACACGTGGGTAACCTACCTATAAGACTGGAATAACTCCGGGAAACCGGGGCTAA-TGCC
Enterococcus_mundtii_BmEM-SD-1_PZ338071           AACACGTGGGTAACCTGCCCATCAGAAGGGGATAACACTTGGAAACAGGTGCTAA-TACC
Staphylococcus_arlettae_ATCC_43957_MH031694       AACACGTGGGTAACCTACCTATAAGACTGGAATAACTCCGGGAAACCGGGGCTAA-TGCC
```

```
KI1_PZ319142                                      GGATAACATATAGAACCGCATGGTTCTATAGTGAAAGATGGTTTTG-CTATCACTTATAG
KI2_PZ319143                                      GGATAACATATAGAACCGCATGGTTCTATAGTGAAAGATGGTTTTG-CTATCACTTATAG
KI3_PZ319144                                      GGATAACATATAGAACCGCATGGTTCTATAGTGAAAGATGGTTTTG-CTATCACTTATAG
KI4_PZ319145                                      GGATAACATATAGAACCGCATGGTTCTATAGTGAAAGATGGTTTTG-CTATCACTTATAG
KI6_PZ319146                                      --------------------TGGTTTCGTTTTGAAAGGCGCTTTACGGTGCCGCTGATGG
KI9_PZ319147                                      GGATAACATATAGAACCGCATGGTTCTATAGTGAAAGATGGTTTTG-CTATCACTTATAG
Staphylococcus_sciuri_SPB_LT745975                GGATAATATTTTGAACCGCATGGTTCAATAGTGAAAGACGGTTTCGGCTGTCACTTATAG
Staphylococcus_xylosus_HG941664                   GGATAACATTTGGAACCGCATGGTTCTAAAGTGAAAGATGGTTTTG-CTATCACTTATAG
Staphylococcus_epidermidis_DSM_20044T_LN681574    GGATAATATATTGAACCGCATGGTTCAATAGTGAAAGACGGTTTTG-CTGTCACTTATAG
Enterococcus_mundtii_LMG_10748_AJ301836           GTATAACAATCGAAACCGCATGGTTTCGTTTTGAAAGGCGCTTTACGGTGCCGCTGATGG
Staphylococcus_gallinarum_KD2_LC782177            GGATAACATATAGAACCGCATGGTTCTATAGTGAAAGATGGTTTTG-CTATCACTTATAG
Enterococcus_mundtii_BmEM-SD-1_PZ338071           GTATAACAATCGAAACCGCATGGTTTCGTTTTGAAAGGCGCTTTACGGTGCCGCTGATGG
Staphylococcus_arlettae_ATCC_43957_MH031694       GGATAACATTTAGAACCGCATGGTTCTAAAGTGAAAGATGGTTTTG-CTATCACTTATAG

KI1_PZ319142                                      ATGGACCCGCGCCGTATTAGCTAGTTGGTAAGGTAATGGCTTACCAAGGCGACGATACGT
KI2_PZ319143                                      ATGGACCCGCGCCGTATTAGCTAGTTGGTAAGGTAATGGCTTACCAAGGCGACGATACGT
KI3_PZ319144                                      ATGGACCCGCGCCGTATTAGCTAGTTGGTAAGGTAATGGCTTACCAAGGCGACGATACGT
KI4_PZ319145                                      ATGGACCCGCGCCGTATTAGCTAGTTGGTAAGGTAATGGCTTACCAAGGCGACGATACGT
KI6_PZ319146                                      ATGGACCCGCGGTGCATTAGCTAGTTGGTGAGGTAACGGCTCACCAAGGCCACGATGCAT
KI9_PZ319147                                      ATGGACCCGCGCCGTATTAGCTAGTTGGTAAGGTAATGGCTTACCAAGGCGACGATACGT
Staphylococcus_sciuri_SPB_LT745975                ATGGACCCGCGCCGTATTAGCTAGTTGGTAAGGTAACGGCTTACCAAGGCGACGATACGT
Staphylococcus_xylosus_HG941664                   ATGGACCCGCGCCGTATTAGCTAGTTGGTAAGGTAACGGCTTACCAAGGCAACGATACGT
```

```
Staphylococcus_epidermidis_DSM_20044T_LN681574  ATGGATCCGCGCCGCATTAGCTAGTTGGTAAGGTAACGGCTTACCAAGGCAACGATGCGT
Enterococcus_mundtii_LMG_10748_AJ301836         ATGGACCCGCGGTGCATTAGCTAGTTGGTGAGGTAACGGCTCACCAAGGCCACGATGCAT
Staphylococcus_gallinarum_KD2_LC782177          ATGGACCCGCGCCGTATTAGCTAGTTGGTAAGGTAATGGCTTACCAAGGCGACGATACGT
Enterococcus_mundtii_BmEM-SD-1_PZ338071         ATGGACCCGCGGTGCATTAGCTAGTTGGTGAGGTAACGGCTCACCAAGGCCACGATGCAT
Staphylococcus_arlettae_ATCC_43957_MH031694     ATGGACCCGCGCCGTATTAGCTAGTTGGTAAGGTAATGGCTTACCAAGGCAACGATACGT

KI1_PZ319142                                    AGCCGACCTGAGAGGGTGATCGGCCACACTGGAACTGAGACACGGTCCAGACTCCTACGG
KI2_PZ319143                                    AGCCGACCTGAGAGGGTGATCGGCCACACTGGAACTGAGACACGGTCCAGACTCCTACGG
KI3_PZ319144                                    AGCCGACCTGAGAGGGTGATCGGCCACACTGGAACTGAGACACGGTCCAGACTCCTACGG
KI4_PZ319145                                    AGCCGACCTGAGAGGGTGATCGGCCACACTGGAACTGAGACACGGTCCAGACTCCTACGG
KI6_PZ319146                                    AGCCGACCTGAGAGGGTGATCGGCCACATTGGGACTGAGACACGGCCCAAACTCCTACGG
KI9_PZ319147                                    AGCCGACCTGAGAGGGTGATCGGCCACACTGGAACTGAGACACGGTCCAGACTCCTACGG
Staphylococcus_sciuri_SPB_LT745975              AGCCGACCTGAGAGGGTGATCGGCCACACTGGAACTGAGACACGGTCCAGACTCCTACGG
Staphylococcus_xylosus_HG941664                 AGCCGACCTGAGAGGGTGATCGGCCACACTGGAACTGAGACACGGTCCAGACTCCTACGG
Staphylococcus_epidermidis_DSM_20044T_LN681574  AGCCGACCTGAGAGGGTGATCGGCCACACTGGAACTGAGACACGGTCCAGACTCCTACGG
Enterococcus_mundtii_LMG_10748_AJ301836         AGCCGACCTGAGAGGGTGATCGGCCACATTGGGACTGAGACACGGCCCAAACTCCTACGG
Staphylococcus_gallinarum_KD2_LC782177          AGCCGACCTGAGAGGGTGATCGGCCACACTGGAACTGAGACACGGTCCAGACTCCTACGG
Enterococcus_mundtii_BmEM-SD-1_PZ338071         AGCCGACCTGAGAGGGTGATCGGCCACATTGGGACTGAGACACGGCCCAAACTCCTACGG
Staphylococcus_arlettae_ATCC_43957_MH031694     AGCCGACCTGAGAGGGTGATCGGCCACACTGGAACTGAGACACGGTCCAGACTCCTACGG

KI1_PZ319142                                    GAGGCAGCAGTAGGGAATCTTCCGCAATGGGCGAAAGCCTGACGGAGCAACGCCGCGTGA
KI2_PZ319143                                    GAGGCAGCAGTAGGGAATCTTCCGCAATGGGCGAAAGCCTGACGGAGCAACGCCGCGTGA
```

KI3_PZ319144	GAGGCAGCAGTAGGGAATCTTCCGCAATGGGCGAAAGCCTGACGGAGCAACGCCGCGTGA
KI4_PZ319145	GAGGCAGCAGTAGGGAATCTTCCGCAATGGGCGAAAGCCTGACGGAGCAACGCCGCGTGA
KI6_PZ319146	GAGGCAGCAGTAGGGAATCTTCGGCAATGGACGAAAGTCTGACCGAGCAACGCCGCGTGA
KI9_PZ319147	GAGGCAGCAGTAGGGAATCTTCCGCAATGGGCGAAAGCCTGACGGAGCAACGCCGCGTGA
Staphylococcus_sciuri_SPB_LT745975	GAGGCAGCAGTAGGGAATCTTCCGCAATGGGCGAAAGCCTGACGGAGCAACGCCGCGTGA
Staphylococcus_xylosus_HG941664	GAGGCAGCAGTAGGGAATCTTCCGCAATGGGCGAAAGCCTGACGGAGCAACGCCGCGTGA
Staphylococcus_epidermidis_DSM_20044T_LN681574	GAGGCAGCAGTAGGGAATCTTCCGCAATGGGCGAAAGCCTGACGGAGCAACGCCGCGTGA
Enterococcus_mundtii_LMG_10748_AJ301836	GAGGCAGCAGTAGGGAATCTTCGGCAATGGACGAAAGTCTGACCGAGCAACGCCGCGTGA
Staphylococcus_gallinarum_KD2_LC782177	GAGGCAGCAGTAGGGAATCTTCCGCAATGGGCGAAAGCCTGACGGAGCAACGCCGCGTGA
Enterococcus_mundtii_BmEM-SD-1_PZ338071	GAGGCAGCAGTAGGGAATCTTCGGCAATGGACGAAAGTCTGACCGAGCAACGCCGCGTGA
Staphylococcus_arlettae_ATCC_43957_MH031694	GAGGCAGCAGTAGGGAATCTTCCGCAATGGGCGAAAGCCTGACGGAGCAACGCCGCGTGA

KI1_PZ319142	GTGATGAAGGGTTTCGGCTCGTAAAACTCTGTTATTAGGGAAGAACATATGTGTAAGTAA
KI2_PZ319143	GTGATGAAGGGTTTCGGCTCGTAAAACTCTGTTATTAGGGAAGAACATATGTGTAAGTAA
KI3_PZ319144	GTGATGAAGGGTTTCGGCTCGTAAAACTCTGTTATTAGGGAAGAACATATGTGTAAGTAA
KI4_PZ319145	GTGATGAAGGGTTTCGGCTCGTAAAACTCTGTTATTAGGGAAGAACATATGTGTAAGTAA
KI6_PZ319146	GTGAAGAAGGTTTTCGGATCGTAAAACTCTGTTGTTAGAGAAGAACAAGGGTGAGAGTAA
KI9_PZ319147	GTGATGAAGGGTTTCGGCTCGTAAAACTCTGTTATTAGGGAAGAACATATGTGTAAGTAA
Staphylococcus_sciuri_SPB_LT745975	GTGATGAAGGTCTTCGGATCGTAAAACTCTGTTGTTAGGGAAGAACAAATTTGTTAGTAA
Staphylococcus_xylosus_HG941664	GTGATGAAGGGTTTCGGCTCGTAAAACTCTGTTATTAGGGAAGAACAAATGTGTAAGTAA
Staphylococcus_epidermidis_DSM_20044T_LN681574	GTGATGAAGGTCTTCGGATCGTAAAACTCTGTTATTAGGGAAGAACAAATGTGTAAGTAA
Enterococcus_mundtii_LMG_10748_AJ301836	GTGAAGAAGGTTTTCGGATCGTAAAACTCTGTTGTTAGAGAAGAACAAGGGTGAGAGTAA

```
Staphylococcus_gallinarum_KD2_LC782177            GTGATGAAGGGTTTCGGCTCGTAAAACTCTGTTATTAGGGAAGAACATATGTGTAAGTAA
Enterococcus_mundtii_BmEM-SD-1_PZ338071           GTGAAGAAGGTTTTCGGATCGTAAAACTCTGTTGTTAGAGAAGAACAAGGGTGAGAGTAA
Staphylococcus_arlettae_ATCC_43957_MH031694       GTGATGAAGGGTTTCGGCTCGTAAAACTCTGTTATTAGGGAAGAACAAACGTGTAAGTAA

KI1_PZ319142                                      CTGTGCACATCTTGACGGTACCTAATCAGAAAGCCACGGCTAACTACGTGCCAGCAGCCG
KI2_PZ319143                                      CTGTGCACATCTTGACGGTACCTAATCAGAAAGCCACGGCTAACTACGTGCCAGCAGCCG
KI3_PZ319144                                      CTGTGCACATCTTGACGGTACCTAATCAGAAAGCCACGGCTAACTACGTGCCAGCAGCCG
KI4_PZ319145                                      CTGTGCACATCTTGACGGTACCTAATCAGAAAGCCACGGCTAACTACGTGCCAGCAGCCG
KI6_PZ319146                                      CTGTTCACCCCTTGACGGTATCTAACCAGAAAGCCACGGCTAACTACGTGCCAGCAGCCG
KI9_PZ319147                                      CTGTGCACATCTTGACGGTACCTAATCAGAAAGCCACGGCTAACTACGTGCCAGCAGCCG
Staphylococcus_sciuri_SPB_LT745975                CTGAACAAGTCTTGACGGTACCTAACCAGAAAGCCACGGCTAACTACGTGCCAGCAGCCG
Staphylococcus_xylosus_HG941664                   CTATGCACATCTTGACGGTACCTAATCAGAAAGCCACGGCTAACTACGTGCCAGCAGCCG
Staphylococcus_epidermidis_DSM_20044T_LN681574    CTATGCACGTCTTGACGGTACCTAATCAGAAAGCCACGGCTAACTACGTGCCAGCAGCCG
Enterococcus_mundtii_LMG_10748_AJ301836           CTGTTCACCCCTTGACGGTATCTAACCAGAAAGCCACGGCTAACTACGTGCCAGCAGCCG
Staphylococcus_gallinarum_KD2_LC782177            CTGTGCACATCTTGACGGTACCTAATCAGAAAGCCACGGCTAACTACGTGCCAGCAGCCG
Enterococcus_mundtii_BmEM-SD-1_PZ338071           CTGTTCACCCCTTGACGGTATCTAACCAGAAAGCCACGGCTAACTACGTGCCAGCAGCCG
Staphylococcus_arlettae_ATCC_43957_MH031694       CTGTGCACGTCTTGACGGTACCTAATCAGAAAGCCACGGCTAACTACGTGCCAGCAGCCG

KI1_PZ319142                                      CGGTAATACGTAGGTGGCAAGCGTTATCCGG-AATTATTGGGCGTAAAGCGCGCGTAGGC
KI2_PZ319143                                      CGGTAATACGTAGGTGGCAAGCGTTATCCGG-AATTATTGGGCGTAAAGCGCGCGTAGGC
KI3_PZ319144                                      CGGTAATACGTAGGTGGCAAGCGTTATCCGG-AATTATTGGGCGTAAAGCGCGCGTAGGC
KI4_PZ319145                                      CGGTAATACGTAGGTGGCAAGCGTTATCCGG-AATTATTGGGCGTAAAGCGCGCGTAGGC
```

KI6_PZ319146	CGGTAATACGTAGGTGGCAAGCGTTGTCCGG-ATTTATTGGGCGTAAAGCGAGCGCAGGC
KI9_PZ319147	CGGTAATACGTAGGTGGCAAGCGTTATCCGG-AATTATTGGGCGTAAAGCGCGCGTAGGC
Staphylococcus_sciuri_SPB_LT745975	CGGTAATACGTAGGTGGCAAGCGTTATCCGGGAATTATTGGGCGTAAAGCGCGCGTAGGC
Staphylococcus_xylosus_HG941664	CGGTAATACGTAGGTGGCAAGCGTTATCCGG-AATTATTGGGCGTAAAGCGCGCGTAGGC
Staphylococcus_epidermidis_DSM_20044T_LN681574	CGGTAATACGTAGGTGGCAAGCGTTATCCGG-AATTATTGGGCGTAAAGCGCGCGTAGGC
Enterococcus_mundtii_LMG_10748_AJ301836	CGGTAATACGTAGGTGGCAAGCGTTGTCCGG-ATTTATTGGGCGTAAAGCGAGCGCAGGC
Staphylococcus_gallinarum_KD2_LC782177	CGGTAATACGTAGGTGGCAAGCGTTATCCGG-AATTATTGGGCGTAAAGCGCGCGTAGGC
Enterococcus_mundtii_BmEM-SD-1_PZ338071	CGGTAATACGTAGGTGGCAAGCGTTGTCCGG-ATTTATTGGGCGTAAAGCGAGCGCAGGC
Staphylococcus_arlettae_ATCC_43957_MH031694	CGGTAATACGTAGGTGGCAAGCGTTATCCGG-AATTATTGGGCGTAAAGCGCGCGTAGGC

KI1_PZ319142	GGTTTCTTAAGTCTGATGTGAAAGCCCACGGCTCAACCGTGGAGGGTCATTGGAAACTGG
KI2_PZ319143	GGTTTCTTAAGTCTGATGTGAAAGCCCACGGCTCAACCGTGGAGGGTCATTGGAAACTGG
KI3_PZ319144	GGTTTCTTAAGTCTGATGTGAAAGCCCACGGCTCAACCGTGGAGGGTCATTGGAAACTGG
KI4_PZ319145	GGTTTCTTAAGTCTGATGTGAAAGCCCACGGCTCAACCGTGGAGGGTCATTGGAAACTGG
KI6_PZ319146	GGTTTCTTAAGTCTGATGTGAAAGCCCCCGGCTCAACCGGGGAGGGTCATTGGAAACTGG
KI9_PZ319147	GGTTTCTTAAGTCTGATGTGAAAGCCCACGGCTCAACCGTGGAGGGTCATTGGAAACTGG
Staphylococcus_sciuri_SPB_LT745975	GGTTTCTTAAGTCTGATGTGAAAGCCCACGGCTCAACCGTGGAGGGTCATTGGAAACTGG
Staphylococcus_xylosus_HG941664	GGTTTCTTAAGTCTGATGTGAAAGCCCACGGCTCAACCGTGGAGGGTCATTGGAAACTGG
Staphylococcus_epidermidis_DSM_20044T_LN681574	GGTTTTTTAAGTCTGATGTGAAAGCCCACGGCTCAACCGTGGAGGGTCATTGGAAACTGG
Enterococcus_mundtii_LMG_10748_AJ301836	GGTTTCTTAAGTCTGATGTGAAAGCCCCCGGCTCAACCGGGGAGGGTCATTGGAAACTGG
Staphylococcus_gallinarum_KD2_LC782177	GGTTTCTTAAGTCTGATGTGAAAGCCCACGGCTCAACCGTGGAGGGTCATTGGAAACTGG
Enterococcus_mundtii_BmEM-SD-1_PZ338071	GGTTTCTTAAGTCTGATGTGAAAGCCCCCGGCTCAACCGGGGAGGGTCATTGGAAACTGG

Staphylococcus_arlettae_ATCC_43957_MH031694	GGTTTCTTAAGTCTGATGTGAAAGCCCACGGCTCAACCGTGGAGGGTCATTGGAAACTGG

KI1_PZ319142	GAAACTTGAGTGCAGAAGAGGAAAGTGGAATTCCATGTGTAGCGGTGAAATGCGCAGAGA
KI2_PZ319143	GAAACTTGAGTGCAGAAGAGGAAAGTGGAATTCCATGTGTAGCGGTGAAATGCGCAGAGA
KI3_PZ319144	GAAACTTGAGTGCAGAAGAGGAAAGTGGAATTCCATGTGTAGCGGTGAAATGCGCAGAGA
KI4_PZ319145	GAAACTTGAGTGCAGAAGAGGAAAGTGGAATTCCATGTGTAGCGGTGAAATGCGCAGAGA
KI6_PZ319146	GAGACTTGAGTGCAGAAGAGGAGAGTGGAATTCCATGTGTAGCGGTGAAATGCGTAGATA
KI9_PZ319147	GAAACTTGAGTGCAGAAGAGGAAAGTGGAATTCCATGTGTAGCGGTGAAATGCGCAGAGA
Staphylococcus_sciuri_SPB_LT745975	GAAACTTGAGTGCAGAAGAGGAGAGTGGAATTCCATGTGTAGCGGTGAAATGCGCAGAGA
Staphylococcus_xylosus_HG941664	GAAACTTGAGTGCAGAAGAGGAAAGTGGAATTCCATGTGTAGCGGTGAAATGCGCAGAGA
Staphylococcus_epidermidis_DSM_20044T_LN681574	AAAACTTGAGTGCAGAAGAGGAAAGTGGAATTCCATGTGTAGCGGTGAAATGCGCAGAGA
Enterococcus_mundtii_LMG_10748_AJ301836	GAGACTTGAGTGCAGAAGAGGAGAGTGGAATTCCATGTGTAGCGGTGAAATGCGTAGATA
Staphylococcus_gallinarum_KD2_LC782177	GAAACTTGAGTGCAGAAGAGGAAAGTGGAATTCCATGTGTAGCGGTGAAATGCGCAGAGA
Enterococcus_mundtii_BmEM-SD-1_PZ338071	GAGACTTGAGTGCAGAAGAGGAGAGTGGAATTCCATGTGTAGCGGTGAAATGCGTAGATA
Staphylococcus_arlettae_ATCC_43957_MH031694	GAGACTTGAGTGCAGAAGAGGAAAGTGGAATTCCATGTGTAGCGGTGAAATGCGCAGAGA

KI1_PZ319142	TATGGAGGAACACCAGTGGCGAAGGCGACTTTCTGGTCTGTAACTGACGCTGATGTGCGA
KI2_PZ319143	TATGGAGGAACACCAGTGGCGAAGGCGACTTTCTGGTCTGTAACTGACGCTGATGTGCGA
KI3_PZ319144	TATGGAGGAACACCAGTGGCGAAGGCGACTTTCTGGTCTGTAACTGACGCTGATGTGCGA
KI4_PZ319145	TATGGAGGAACACCAGTGGCGAAGGCGACTTTCTGGTCTGTAACTGACGCTGATGTGCGA
KI6_PZ319146	TATGGAGGAACACCAGTGGCGAAGGCGGCTCTCTGGTCTGTAACTGACGCTGAGGCTCGA
KI9_PZ319147	TATGGAGGAACACCAGTGGCGAAGGCGACTTTCTGGTCTGTAACTGACGCTGATGTGCGA

Staphylococcus_sciuri_SPB_LT745975	TATGGAGGAACACCAGTGGCGAAGGCGGCTCTCTGGTCTGTAACTGACGCTGATGTGCGA
Staphylococcus_xylosus_HG941664	TATGGAGGAACACCAGTGGCGAAGGCGACTTTCTGGTCTGTAACTGACGCTGATGTGCGA
Staphylococcus_epidermidis_DSM_20044T_LN681574	TATGGAGGAACACCAGTGGCGAAGGCGACTTTCTGGTCTGTAACTGACGCTGATGTGCGA
Enterococcus_mundtii_LMG_10748_AJ301836	TATGGAGGAACACCAGTGGCGAAGGCGGCTCTCTGGTCTGTAACTGACGCTGAGGCTCGA
Staphylococcus_gallinarum_KD2_LC782177	TATGGAGGAACACCAGTGGCGAAGGCGACTTTCTGGTCTGTAACTGACGCTGATGTGCGA
Enterococcus_mundtii_BmEM-SD-1_PZ338071	TATGGAGGAACACCAGTGGCGAAGGCGGCTCTCTGGTCTGTAACTGACGCTGAGGCTCGA
Staphylococcus_arlettae_ATCC_43957_MH031694	TATGGAGGAACACCAGTGGCGAAGGCGACTTTCTGGTCTGTAACTGACGCTGATGTGCGA

KI1_PZ319142	AAGCGTGGGGATCAAACAGGATTAGATACCCTGGTAGTCCACGCCGTAAACGATGAGTGC
KI2_PZ319143	AAGCGTGGGGATCAAACAGGATTAGATACCCTGGTAGTCCACGCCGTAAACGATGAGTGC
KI3_PZ319144	AAGCGTGGGGATCAAACAGGATTAGATACCCTGGTAGTCCACGCCGTAAACGATGAGTGC
KI4_PZ319145	AAGCGTGGGGATCAAACAGGATTAGATACCCTGGTAGTCCACGCCGTAAACGATGAGTGC
KI6_PZ319146	AAGCGTGGGGAGCAAACAGGATTAGATACCCTGGTAGTCCACGCCGTAAACGATGAGTGC
KI9_PZ319147	AAGCGTGGGGATCAAACAGGATTAGATACCCTGGTAGTCCACGCCGTAAACGATGAGTGC
Staphylococcus_sciuri_SPB_LT745975	AAGCGTGGGGATCAAACAGGATTAGATACCCTGGTAGTCCACGCCGTAAACGATGAGTGC
Staphylococcus_xylosus_HG941664	AAGCGTGGGGATCAAACAGGATTAGATACCCTGGTAGTCCACGCCGTAAACGATGAGTGC
Staphylococcus_epidermidis_DSM_20044T_LN681574	AAGCGTGGGGATCAAACAGGATTAGATACCCTGGTAGTCCACGCCGTAAACGATGAGTGC
Enterococcus_mundtii_LMG_10748_AJ301836	AAGCGTGGGG-GCAAACAGGATTAGATACCCTGGTAGTCCACGCCGTAAACGATGAGTGC
Staphylococcus_gallinarum_KD2_LC782177	AAGCGTGGGGATCAAACAGGATTAGATACCCTGGTAGTCCACGCCGTAAACGATGAGTGC
Enterococcus_mundtii_BmEM-SD-1_PZ338071	AAGCGTGGGGAGCAAACAGGATTAGATACCCTGGTAGTCCACGCCGTAAACGATGAGTGC
Staphylococcus_arlettae_ATCC_43957_MH031694	AAGCGTGGGGATCAAACAGGATTAGATACCCTGGTAGTCCACGCCGTAAACGATGAGTGC

```
KI1_PZ319142                                   TAAGTGTTAGGGGGTTTCCGCCCCTTAGTGCTGCAGCT-AACGCATTAAGCACTCCGCCT
KI2_PZ319143                                   TAAGTGTTAGGGGGTTTCCGCCCCTTAGTGCTGCAGCT-AACGCATTAAGCACTCCGCCT
KI3_PZ319144                                   TAAGTGTTAGGGGGTTTCCGCCCCTTAGTGCTGCAGCT-AACGCATTAAGCACTCCGCCT
KI4_PZ319145                                   TAAGTGTTAGGGGGTTTCCGCCCCTTAGTGCTGCAGCT-AACGCATTAAGCACTCCGCCT
KI6_PZ319146                                   TAAGTGTTGGAGGGTTTCCGCCCTTCAGTGCTGCAGCT-AACGCATTAAGCACTCCGCCT
KI9_PZ319147                                   TAAGTGTTAGGGGGTTTCCGCCCCTTAGTGCTGCAGCT-AACGCATTAAGCACTCCGCCT
Staphylococcus_sciuri_SPB_LT745975             TAAGTGTTAGGGGGTTTCCGCCCCTTAGTGCTGCAGCT-AACGCATTAAGCACTCCGCCT
Staphylococcus_xylosus_HG941664                TAAGTGTTAGGGGGTTTCCGCCCCTTAGTGCTGCAGCT-AACGCATTAAGCACTCCGCCT
Staphylococcus_epidermidis_DSM_20044T_LN681574 TAAGTGTTAGGGGGTTTCCGCCCCTTAGTGCTGCAGCT-AACGCATTAAGCACTCCGCCT
Enterococcus_mundtii_LMG_10748_AJ301836        TAAGTGTTGGAGGGTTTCCGCCCTTCAGTGCTGCAGCT-AACGCATTAAGCACTCCGCCT
Staphylococcus_gallinarum_KD2_LC782177         TAAGTGTTAGGGGGTTTCCGCCCCTTAGTGCTGCAGCT-AACGCATTAAGCACTCCGCCT
Enterococcus_mundtii_BmEM-SD-1_PZ338071        TAAGTGTTGGAGGGTTTCCGCCCTTCAGTGCTGCAGCT-AACGCATTAAGCACTCCGCCT
Staphylococcus_arlettae_ATCC_43957_MH031694    TAAGTGTTAGGGGGTTTCCGCCCCTTAGTGCTGCAGCTTAACGGCCTAAGCACTCCGCCT

KI1_PZ319142                                   GGGGAGTACGACCGCAAGGTTGAAACTCAAAGGAATTGACGGGGACCCGCACAAGCGGTG
KI2_PZ319143                                   GGGGAGTACGACCGCAAGGTTGAAACTCAAAGGAATTGACGGGGACCCGCACAAGCGGTG
KI3_PZ319144                                   GGGGAGTACGACCGCAAGGTTGAAACTCAAAGGAATTGACGGGGACCCGCACAAGCGGTG
KI4_PZ319145                                   GGGGAGTACGACCGCAAGGTTGAAACTCAAAGGAATTGACGGGGACCCGCACAAGCGGTG
KI6_PZ319146                                   GGGGAGTACGACCGCAAGGTTGAAACTCAAAGGAATTGACGGGGGCCCGCACAAGCGGTG
KI9_PZ319147                                   GGGGAGTACGACCGCAAGGTTGAAACTCAAAGGAATTGACGGGGACCCGCACAAGCGGTG
Staphylococcus_sciuri_SPB_LT745975             GGGGAGTACGACCGCAAGGTTGAAACTCAAAGGAATTGACGGGGACCCGCACAAGCGGTG
Staphylococcus_xylosus_HG941664                GGGGAGTACGACCGCAAGGTTGAAACTCAAAGGAATTGACGGGGACCCGCACAAGCGGTG
```

Staphylococcus_epidermidis_DSM_20044T_LN681574	GGGGAGTACGACCGCAAGGTTGAAACTCAAAGGAATTGACGGGGACCCGCACAAGCGGTG
Enterococcus_mundtii_LMG_10748_AJ301836	GGGGAGTACGACCGCAAGGTTGAAACTCAAAGGAATTGACGGGGGCCCGCACAAGCGGTG
Staphylococcus_gallinarum_KD2_LC782177	GGGGAGTACGACCGCAAGGTTGAAACTCAAAGGAATTGACGGGGACCCGCACAAGCGGTG
Enterococcus_mundtii_BmEM-SD-1_PZ338071	GGGGAGTACGACCGCAAGGTTGAAACTCAAAGGAATTGACGGGGGCCCGCACAAGCGGTG
Staphylococcus_arlettae_ATCC_43957_MH031694	GGGGAGTACGACCGCAAGGTTGAAACTCAAAGGAATTGACGGGGACCCGCACAAGCGGTG

KI1_PZ319142	GAGCATGTGGTTTAATTCGAAGCAACGCGAAGAACCTTACCAAATCTTGACATCCTTTGA
KI2_PZ319143	GAGCATGTGGTTTAATTCGAAGCAACGCGAAGAACCTTACCAAATCTTGACATCCTTTGA
KI3_PZ319144	GAGCATGTGGTTTAATTCGAAGCAACGCGAAGAACCTTACCAAATCTTGACATCCTTTGA
KI4_PZ319145	GAGCATGTGGTTTAATTCGAAGCAACGCGAAGAACCTTACCAAATCTTGACATCCTTTGA
KI6_PZ319146	GAGCATGTGGTTTAATTCGAAGCAACGCGAAGAACCTTACCAGGTCTTGACATCCTTTGA
KI9_PZ319147	GAGCATGTGGTTTAATTCGAAGCAACGCGAAGAACCTTACCAAATCTTGACATCCTTTGA
Staphylococcus_sciuri_SPB_LT745975	GAGCATGTGGTTTAATTCGAAGCAACGCGAAGAACCTTACCAAATCTTGACATCCTTTGA
Staphylococcus_xylosus_HG941664	GAGCATGTGGTTTAATTCGAAGCAACGCGAAGAACCTTACCAAATCTTGACATCCTTTGA
Staphylococcus_epidermidis_DSM_20044T_LN681574	GAGCATGTGGTTTAATTCGAAGCAACGCGAAGAACCTTACCAAATCTTGACATCCTCTGA
Enterococcus_mundtii_LMG_10748_AJ301836	GAGCATGTGGTTTAATTCGAAGCAACGCGAAGAACCTTACCAGGTCTTGACATCCTTTGA
Staphylococcus_gallinarum_KD2_LC782177	GAGCATGTGGTTTAATTCGAAGCAACGCGAAGAACCTTACCAAATCTTGACATCCTTTGA
Enterococcus_mundtii_BmEM-SD-1_PZ338071	GAGCATGTGGTTTAATTCGAAGCAACGCGAAGAACCTTACCAGGTCTTGACATCCTTTGA
Staphylococcus_arlettae_ATCC_43957_MH031694	GAGCATGTGGTTTAATTCGAAGCAACGCGAAGAACCTTACCAAATCTTGACATCCTTTGA

KI1_PZ319142	CCACTCTAGAGATAGAGCTTTCCCCTTCGGGGGACAAAGTGACAGGTGGTGCATGGTTGT
KI2_PZ319143	CCACTCTAGAGATAGAGCTTTCCCCTTCGGGGGACAAAGTGACAGGTGGTGCATGGTTGT

KI3_PZ319144	CCACTCTAGAGATAGAGCTTTCCCCTTCGGGGGACAAAGTGACAGGTGGTGCATGGTTGT
KI4_PZ319145	CCACTCTAGAGATAGAGCTTTCCCCTTCGGGGGACAAAGTGACAGGTGGTGCATGGTTGT
KI6_PZ319146	CCACTCTAGAGATAGAG-CTTCCCCTTCGGGGG-CAAAGTGACAGGTGGTGCATGGTTGT
KI9_PZ319147	CCACTCTAGAGATAGAGCTTTCCCCTTCGGGGGACAAAGTGACAGGTGGTGCATGGTTGT
Staphylococcus_sciuri_SPB_LT745975	CCGCTCTAGAGATAGAGTCTTCCCCTTCGGGGGACAAAGTGACAGGTGGTGCATGGTTGT
Staphylococcus_xylosus_HG941664	AAACTCTAGAGATAGAGCCTTCCCCTTCGGGGGACAAAGTGACAGGTGGTGCATGGTTGT
Staphylococcus_epidermidis_DSM_20044T_LN681574	CCCCTCTAGAGATAGAGTTTTCCCCTTCGGGGGACAGAGTGACAGGTGGTGCATGGTTGT
Enterococcus_mundtii_LMG_10748_AJ301836	CCACTCTAGAGATAGAG-CTTCCCCTTCGGGGG-CAAAGTGACAGGTGGTGCATGGTTGG
Staphylococcus_gallinarum_KD2_LC782177	CCACTCTAGAGATAGAGCTTTCCCCTTCGGGGGACAAAGTGACAGGTGGTGCATGGTTGT
Enterococcus_mundtii_BmEM-SD-1_PZ338071	CCACTCTAGAGATAGAG-CTTCCCCTTCGGGGG-CAAAGTGACAGGTGGTGCATGGTTGT
Staphylococcus_arlettae_ATCC_43957_MH031694	CCACTCTAGAGATAGAGCTTTCCCCTTCGGGGGACAAAGTGACAGGTGGTGCATGGTTGT

KI1_PZ319142	CGTCAGCTCGTGTCGTGAGATGTTGGGTTAAGTCCCGCAACGAGCGCAACCCTTAAGCTT
KI2_PZ319143	CGTCAGCTCGTGTCGTGAGATGTTGGGTTAAGTCCCGCAACGAGCGCAACCCTTAAGCTT
KI3_PZ319144	CGTCAGCTCGTGTCGTGAGATGTTGGGTTAAGTCCCGCAACGAGCGCAACCCTTAAGCTT
KI4_PZ319145	CGTCAGCTCGTGTCGTGAGATGTTGGGTTAAGTCCCGCAACGAGCGCAACCCTTAAGCTT
KI6_PZ319146	CGTCAGCTCGTGTCGTGAGATGTTGGGTTAAGTCCCGCAACGAGCGCAACCCTTATTGTT
KI9_PZ319147	CGTCAGCTCGTGTCGTGAGATGTTGGGTTAAGTCCCGCAACGAGCGCAACCCTTAAGCTT
Staphylococcus_sciuri_SPB_LT745975	CGTCAGCTCGTGTCGTGAGATGTTGGGTTAAGTCCCGCAACGAGCGCAACCCTTAAGCTT
Staphylococcus_xylosus_HG941664	CGTCAGCTCGTGTCGTGAGATGTTGGGTTAAGTCCCGCAACGAGCGCAACCCTTAAGCTT
Staphylococcus_epidermidis_DSM_20044T_LN681574	CGTCAGCTCGTGTCGTGAGATGTTGGGTTAAGTCCCGCAACGAGCGCAACCCTTAAGCTT
Enterococcus_mundtii_LMG_10748_AJ301836	CGTCAGCTCGTGTCGTGAGATGTTGGGTTAAGTCCCGCAACGAGCGCAACCCTTATTGTT

Staphylococcus_gallinarum_KD2_LC782177	CGTCAGCTCGTGTCGTGAGATGTTGGGTTAAGTCCCGCAACGAGCGCAACCCTTAAGCTT
Enterococcus_mundtii_BmEM-SD-1_PZ338071	CGTCAGCTCGTGTCGTGAGATGTTGGGTTAAGTCCCGCAACGAGCGCAACCCTTATTGTT
Staphylococcus_arlettae_ATCC_43957_MH031694	CGTCAGCTCGTGTCGTGAGATGTTGGGTTAAGTCCCGCAACGAGCGCAACCCTTAAACTT
KI1_PZ319142	AGTTGCCATCATTAAGTTGGGCACTCTAGGTTGACTGCCGGTGACAAACCGGAGGAAGGT
KI2_PZ319143	AGTTGCCATCATTAAGTTGGGCACTCTAGGTTGACTGCCGGTGACAAACCGGAGGAAGGT
KI3_PZ319144	AGTTGCCATCATTAAGTTGGGCACTCTAGGTTGACTGCCGGTGACAAACCGGAGGAAGGT
KI4_PZ319145	AGTTGCCATCATTAAGTTGGGCACTCTAGGTTGACTGCCGGTGACAAACCGGAGGAAGGT
KI6_PZ319146	AGTTGCCATCATTTAGTTGGGCACTCTAGCAAGACTGCCGGTGACAAACCGGAGGAAGGT
KI9_PZ319147	AGTTGCCATCATTAAGTTGGGCACTCTAGGTTGACTGCCGGTGACAAACCGGAGGAAGGT
Staphylococcus_sciuri_SPB_LT745975	AGTTGCCATCATTAAGTTGGGCACTCTAGGTTGACTGCCGGTGACAAACCGGAGGAAGGT
Staphylococcus_xylosus_HG941664	AGTTGCCATCATTAAGTTGGGCACTCTAGGTTGACTGCCGGTGACAAACCGGAGGAAGGT
Staphylococcus_epidermidis_DSM_20044T_LN681574	AGTTGCCATCATTAAGTTGGGCACTCTAAGTTGACTGCCGGTGACAAACCGGAGGAAGGT
Enterococcus_mundtii_LMG_10748_AJ301836	AGTTGCCATCATTTAGTTGGGCACTCTAGCAAGACTGCCGGTGACAAACCGGAGGAAGGT
Staphylococcus_gallinarum_KD2_LC782177	AGTTGCCATCATTAAGTTGGGCACTCTAGGTTGACTGCCGGTGACAAACCGGAGGAAGGT
Enterococcus_mundtii_BmEM-SD-1_PZ338071	AGTTGCCATCATTTAGTTGGGCACTCTAGCAAGACTGCCGGTGACAAACCGGAGGAAGGT
Staphylococcus_arlettae_ATCC_43957_MH031694	AGTTGCCAGCATTTAGTTGGGCACTCTAGGTTGACTGCCGGTGACAAACCGGAGGAAGGT
KI1_PZ319142	GGGGATGACGTCAAATCATCATGCCCCTTATGATTTGGGCTACACACGTGCTACAATGGA
KI2_PZ319143	GGGGATGACGTCAAATCATCATGCCCCTTATGATTTGGGCTACACACGTGCTACAATGGA
KI3_PZ319144	GGGGATGACGTCAAATCATCATGCCCCTTATGATTTGGGCTACACACGTGCTACAATGGA
KI4_PZ319145	GGGGATGACGTCAAATCATCATGCCCCTTATGATTTGGGCTACACACGTGCTACAATGGA

```
KI6_PZ319146                                  GGGGATGACGTCAAATCATCATGCCCCTTATGACCTGGGCTACACACGTGCTACAATGGG
KI9_PZ319147                                  GGGGATGACGTCAAATCATCATGCCCCTTATGATTTGGGCTACACACGTGCTACAATGGA
Staphylococcus_sciuri_SPB_LT745975            GGGGATGACGTCAAATCATCATGCCCCTTATGATTTGGGCTACACACGTGCTACAATGGA
Staphylococcus_xylosus_HG941664               GGGGATGACGTCAAATCATCATGCCCCTTATGATTTGGGCTACACACGTGCTACAATGGA
Staphylococcus_epidermidis_DSM_20044T_LN681574 GGGGATGACGTCAAATCATCATGCCCCTTATGATTTGGGCTACACACGTGCTACAATGGA
Enterococcus_mundtii_LMG_10748_AJ301836       GGGGATGACGTCAAATCATCATGCCCCTTATGACCTGGGCTACACACGTGCTACAATGGG
Staphylococcus_gallinarum_KD2_LC782177        GGGGATGACGTCAAATCATCATGCCCCTTATGATTTGGGCTACACACGTGCTACAATGGA
Enterococcus_mundtii_BmEM-SD-1_PZ338071       GGGGATGACGTCAAATCATCATGCCCCTTATGACCTGGGCTACACACGTGCTACAATGGG
Staphylococcus_arlettae_ATCC_43957_MH031694   GGGGATGACGTCAAATCATCATGCCCCTTATGATTTGGGCTACACACGTGCTACAATGGA

KI1_PZ319142                                  CAATACAAAGGGCAGCTAAACCGTGAGGTCATGCAAATCCCATAAAGTTGTTCTCAGTTC
KI2_PZ319143                                  CAATACAAAGGGCAGCTAAACCGTGAGGTCATGCAAATCCCATAAAGTTGTTCTCAGTTC
KI3_PZ319144                                  CAATACAAAGGGCAGCTAAACCGCGAGGTCATGCAAATCCCATAAAGTTGTTCTCAGTTC
KI4_PZ319145                                  CAATACAAAGGGCAGCTAAACCGCGAGGTCATGCAAATCCCATAAAGTTGTTCTCAGTTC
KI6_PZ319146                                  AAGTACAACGAGTCGCGAAGTCGCGAGGCTAAGCTAATCTCTTAAAGCTTCTCTCAGTTC
KI9_PZ319147                                  CAATACAAAGGGCAGCTAAACCGCGAGGTCATGCAAATCCCATAAAGTTGTTCTCAGTTC
Staphylococcus_sciuri_SPB_LT745975            TAATACAAAGGGCAGCGAATCCGCGAGGCCAAGCAAATCCCATAAAATTATTCTCAGTTC
Staphylococcus_xylosus_HG941664               CAATACAAAGGGCAGCTAAACCGCGAGGTCATGCAAATCCCATAAAGTTGTTCTCAGTTC
Staphylococcus_epidermidis_DSM_20044T_LN681574 CAATACAAAGGGCAGCGAAACCGCGAGGTCAAGCAAATCCCATAAAGTTGTTCTCAGTTC
Enterococcus_mundtii_LMG_10748_AJ301836       AAGTACAACGAGTCGCGAAGTCGCGAGGCTAAGCTAATCTCTTAAAGCTTCTCTCAGTTC
Staphylococcus_gallinarum_KD2_LC782177        CAATACAAAGGGCAGCTAAACCGCGAGGTCATGCAAATCCCATAAAGTTGTTCTCAGTTC
Enterococcus_mundtii_BmEM-SD-1_PZ338071       AAGTACAACGAGTCGCGAAGTCGCGAGGCTAAGCTAATCTCTTAAAGCTTCTCTCAGTTC
```

```
Staphylococcus_arlettae_ATCC_43957_MH031694      CAATACAAAGGGCAGCTAAACCGCGAGGTCATGCAAATCCCATAAAGTTGTTCTCAGTTC

KI1_PZ319142                                     GGATTGTAGTCTGCAACTCGACTACATGAAGCTGGAATCGCTAGTAATCGTAGATCAGCA
KI2_PZ319143                                     GGATTGTAGTCTGCAACTCGACTACATGAAGCTGGAATCGCTAGTAATCGTAGATCAGCA
KI3_PZ319144                                     GGATTGTAGTCTGCAACTCGACTACATGAAGCTGGAATCGCTAGTAATCGTAGATCAGCA
KI4_PZ319145                                     GGATTGTAGTCTGCAACTCGACTACATGAAGCTGGAATCGCTAGTAATCGTAGATCAGCA
KI6_PZ319146                                     GGATTGTAGGCTGCAACTCGCCTACATGAAGCCGGAATCGCTAGTAATCGCGGATCAGCA
KI9_PZ319147                                     GGATTGTAGTCTGCAACTCGACTACATGAAGCTGGAATCGCTAGTAATCGTAGATCAGCA
Staphylococcus_sciuri_SPB_LT745975               GGATTGTAGTCTGCAACTCGACTACATGAAGCTGGAATCGCTAGTAATCGTAGATCAGCA
Staphylococcus_xylosus_HG941664                  GGATTGTAGTCTGCAACTCGACTACATGAAGCTGGAATCGCTAGTAATCGTAGATCAGCA
Staphylococcus_epidermidis_DSM_20044T_LN681574   GGATTGTAGTCTGCAACTCGACTATATGAAGCTGGAATCGCTAGTAATCGTAGATCAGCA
Enterococcus_mundtii_LMG_10748_AJ301836          GGATTGTAGGCTGCAACTCGCCTACATGAAGCCGGAATCGCTAGTAATCGCGGATCAGCA
Staphylococcus_gallinarum_KD2_LC782177           GGATTGTAGTCTGCAACTCGACTACATGAAGCTGGAATCGCTAGTAATCGTAGATCAGCA
Enterococcus_mundtii_BmEM-SD-1_PZ338071          GGATTGTAGGCTGCAACTCGCCTACATGAAGCCGGAATCGCTAGTAATCGCGGATCAGCA
Staphylococcus_arlettae_ATCC_43957_MH031694      GGATTGTAGTCTGCAACTCGACTACATGAAGCTGGAATCGCTAGTAATCGTAGATCAGCA

KI1_PZ319142                                     TGCTACGGTGAATACGTTCCCGGGTCTTGTACACACCGCCCGTCACACCACGAG-AGTTT
KI2_PZ319143                                     TGCTACGGTGAATACGTTCCCGGGTCTTGTACACACCGCCCGTCACACCACGAG-AGTTT
KI3_PZ319144                                     TGCTACGGTGAATACGTTCCCGGGTCTTGTACACACCGCCCGTCACACCACGAG-AGTTT
KI4_PZ319145                                     TGCTACGGTGAATACGTTCCCGGGTCTTGTACACACCGCCCGTCACACCACGAG-AGTTT
KI6_PZ319146                                     CGCCGCGGTGAATACGTTCCCGGGCCTTGTACACACCGCCCGTCACACCACGAG-AGTTT
KI9_PZ319147                                     TGCTACGGTGAATACGTTCCCGGGTCTTGTACACACCGCCCGTCACACCACGAG-AGTTT
```

```
Staphylococcus_sciuri_SPB_LT745975           TGCTACGGTGAATACGTTCCCGGGTCTTGTACACACCGCCCGTCACACCACGAG-AGTTT
Staphylococcus_xylosus_HG941664              TGCTACGGTGAATACGTTCCCGGGTCTTGTACACACCGCCCGTCACACCACGAG-AGTTT
Staphylococcus_epidermidis_DSM_20044T_LN681574 TGCTACGGTGAATACGTTCCCGGGTCTTGTACACACCGCCCGTCACACCACGAG-AGTTT
Enterococcus_mundtii_LMG_10748_AJ301836      CGCCGCGGTGAATACGTTCCCGGGCCTTGTACACACCGCCCGTCACACCACGAGCAGTTT
Staphylococcus_gallinarum_KD2_LC782177       TGCTACGGTGAATACGTTCCCGGGTCTTGTACACACCGCCCGTCACACCACGAG-AGTTT
Enterococcus_mundtii_BmEM-SD-1_PZ338071      CGCCGCGGTGAATACGTTCCCGGGCCTTGTACACACCGCCCGTCACACCACGAG-AGTTT
Staphylococcus_arlettae_ATCC_43957_MH031694  TGCTACGGTGAATACGTTCCCGGGTCTTGTACACACCGCCCGTCACACCACGAG-AGTTT

KI1_PZ319142                                 GTAACACCCGAAG-CCGGTGGAGTAACCATTTATGGAGCTAG------------------
KI2_PZ319143                                 GTAACACCCGAAG-CCGGTGGAGTAACCATTTA---------------------------
KI3_PZ319144                                 GTAACACCCGAAG-CCGGTGGAGTAACCATTTATGGAGCTAGC-----------------
KI4_PZ319145                                 GTAACACCCGAAG-CCGGTGGAGTAACCATTTA---------------------------
KI6_PZ319146                                 GTAACACCCGAAG-TCGGTGAG-GTAAC--------------------------------
KI9_PZ319147                                 GTAACACCCGAAG-CCGGTGGAGTAACCATTTA---------------------------
Staphylococcus_sciuri_SPB_LT745975           GTA-CACCCGAAG-CCG-------------------------------------------
Staphylococcus_xylosus_HG941664              GTAACACCCGAAG-CCGGTGGAGTAACCATTTATGGAGCTAGCCGTCGAAGGTGGGACAA
Staphylococcus_epidermidis_DSM_20044T_LN681574 GTAACACCCGAAG-CCGGTGGAGTAACCATTT--GGAGCTAGCCGTCGAAGGTGGGACAA
Enterococcus_mundtii_LMG_10748_AJ301836      GTAACACCCGAAGCTCGGTGAGCGTAACCTTTTTGGAGCCAGCCGCCTAAGGTGGGATAG
Staphylococcus_gallinarum_KD2_LC782177       GTAACACCCGAAG-CCGGTGGAGTAACCATTTATGGAGCTAGCCGTCGAA----------
Enterococcus_mundtii_BmEM-SD-1_PZ338071      GTAACACCCGAAG-TCGGTGAG-GTAACCTTTTTGGAGCCAGCCGCCTAA----------
Staphylococcus_arlettae_ATCC_43957_MH031694  GTAACACCCGAAG-CCGGTGGAGTAACCATTTATGGAGCTAGCCGTCAAAGGTGGGACAA
```

```
KI1_PZ319142                                       ------------------------------------------------------------
KI2_PZ319143                                       ------------------------------------------------------------
KI3_PZ319144                                       ------------------------------------------------------------
KI4_PZ319145                                       ------------------------------------------------------------
KI6_PZ319146                                       ------------------------------------------------------------
KI9_PZ319147                                       ------------------------------------------------------------
Staphylococcus_sciuri_SPB_LT745975                 ------------------------------------------------------------
Staphylococcus_xylosus_HG941664                    ATGATTGGGGTGAAGTCGTAACAAGGTAGCCGTA--------------------------
Staphylococcus_epidermidis_DSM_20044T_LN681574     ATGATTGGGGTGAAGTCGTAACAAGGTAGCCGTATCGGAAGG------------------
Enterococcus_mundtii_LMG_10748_AJ301836            ATGATTGGGGTGAAGTCGTAACAAGGTAGCCGTATCGAAGGTGCGGCTGGATCACCTCCT
Staphylococcus_gallinarum_KD2_LC782177             ------------------------------------------------------------
Enterococcus_mundtii_BmEM-SD-1_PZ338071            ------------------------------------------------------------
Staphylococcus_arlettae_ATCC_43957_MH031694        ATGATTGGGGTGAAGTCTTAACAAGGGTACCGTAAA------------------------

KI1_PZ319142                                       ------------------------------------------------------------
KI2_PZ319143                                       ------------------------------------------------------------
KI3_PZ319144                                       ------------------------------------------------------------
KI4_PZ319145                                       ------------------------------------------------------------
KI6_PZ319146                                       ------------------------------------------------------------
KI9_PZ319147                                       ------------------------------------------------------------
Staphylococcus_sciuri_SPB_LT745975                 ------------------------------------------------------------
Staphylococcus_xylosus_HG941664                    ------------------------------------------------------------
```

```
Staphylococcus_epidermidis_DSM_20044T_LN681574  ------------------------------------------------------------
Enterococcus_mundtii_LMG_10748_AJ301836         TTCTAAGGAATATTACGGAGACTACACACGTTTGTCGATACTTTGTTCAGTTTTGAGAGG
Staphylococcus_gallinarum_KD2_LC782177          ------------------------------------------------------------
Enterococcus_mundtii_BmEM-SD-1_PZ338071         ------------------------------------------------------------
Staphylococcus_arlettae_ATCC_43957_MH031694     ------------------------------------------------------------

KI1_PZ319142                                    ------------------------------------------------------------
KI2_PZ319143                                    ------------------------------------------------------------
KI3_PZ319144                                    ------------------------------------------------------------
KI4_PZ319145                                    ------------------------------------------------------------
KI6_PZ319146                                    ------------------------------------------------------------
KI9_PZ319147                                    ------------------------------------------------------------
Staphylococcus_sciuri_SPB_LT745975              ------------------------------------------------------------
Staphylococcus_xylosus_HG941664                 ------------------------------------------------------------
Staphylococcus_epidermidis_DSM_20044T_LN681574  ------------------------------------------------------------
Enterococcus_mundtii_LMG_10748_AJ301836         TCTACTCTCAATATTTTGTTCATTGATAACTGGATATTGAGTAGACATGTATGTATACAG
Staphylococcus_gallinarum_KD2_LC782177          ------------------------------------------------------------
Enterococcus_mundtii_BmEM-SD-1_PZ338071         ------------------------------------------------------------
Staphylococcus_arlettae_ATCC_43957_MH031694     ------------------------------------------------------------

KI1_PZ319142                                    ------------------------------------------------------------
KI2_PZ319143                                    ------------------------------------------------------------
```

```
KI3_PZ319144                                     ------------------------------------------------------------
KI4_PZ319145                                     ------------------------------------------------------------
KI6_PZ319146                                     ------------------------------------------------------------
KI9_PZ319147                                     ------------------------------------------------------------
Staphylococcus_sciuri_SPB_LT745975               ------------------------------------------------------------
Staphylococcus_xylosus_HG941664                  ------------------------------------------------------------
Staphylococcus_epidermidis_DSM_20044T_LN681574   ------------------------------------------------------------
Enterococcus_mundtii_LMG_10748_AJ301836          AGCGCAGAACACCGCGTTGCTGAGTTTTTTAATAAATTCATTGCTTATATCTTGACTCAT
Staphylococcus_gallinarum_KD2_LC782177           ------------------------------------------------------------
Enterococcus_mundtii_BmEM-SD-1_PZ338071          ------------------------------------------------------------
Staphylococcus_arlettae_ATCC_43957_MH031694      ------------------------------------------------------------

KI1_PZ319142                                     ------------------------------------------------------------
KI2_PZ319143                                     ------------------------------------------------------------
KI3_PZ319144                                     ------------------------------------------------------------
KI4_PZ319145                                     ------------------------------------------------------------
KI6_PZ319146                                     ------------------------------------------------------------
KI9_PZ319147                                     ------------------------------------------------------------
Staphylococcus_sciuri_SPB_LT745975               ------------------------------------------------------------
Staphylococcus_xylosus_HG941664                  ------------------------------------------------------------
Staphylococcus_epidermidis_DSM_20044T_LN681574   ------------------------------------------------------------
Enterococcus_mundtii_LMG_10748_AJ301836          CGTAGAGAGAGATTAACCACGTAGTCGTGAGGTGAGTGATAGGCGACGTGATGCTTGCTC
```

```
Staphylococcus_gallinarum_KD2_LC782177          ------------------------------------------------------------
Enterococcus_mundtii_BmEM-SD-1_PZ338071         ------------------------------------------------------------
Staphylococcus_arlettae_ATCC_43957_MH031694     ------------------------------------------------------------

KI1_PZ319142                                    ------------------------------------------------------------
KI2_PZ319143                                    ------------------------------------------------------------
KI3_PZ319144                                    ------------------------------------------------------------
KI4_PZ319145                                    ------------------------------------------------------------
KI6_PZ319146                                    ------------------------------------------------------------
KI9_PZ319147                                    ------------------------------------------------------------
Staphylococcus_sciuri_SPB_LT745975              ------------------------------------------------------------
Staphylococcus_xylosus_HG941664                 ------------------------------------------------------------
Staphylococcus_epidermidis_DSM_20044T_LN681574  ------------------------------------------------------------
Enterococcus_mundtii_LMG_10748_AJ301836         TATGAGCGATGAGGATGGACTATACGATATGTTGACGAACTCTACGTACATGATCAGATA
Staphylococcus_gallinarum_KD2_LC782177          ------------------------------------------------------------
Enterococcus_mundtii_BmEM-SD-1_PZ338071         ------------------------------------------------------------
Staphylococcus_arlettae_ATCC_43957_MH031694     ------------------------------------------------------------

KI1_PZ319142                                    -----------
KI2_PZ319143                                    -----------
KI3_PZ319144                                    -----------
KI4_PZ319145                                    -----------
```

```
KI6_PZ319146                                 -----------
KI9_PZ319147                                 -----------
Staphylococcus_sciuri_SPB_LT745975           -----------
Staphylococcus_xylosus_HG941664              -----------
Staphylococcus_epidermidis_DSM_20044T_LN681574  -----------
Enterococcus_mundtii_LMG_10748_AJ301836      TTTCGTGCGAA
Staphylococcus_gallinarum_KD2_LC782177       -----------
Enterococcus_mundtii_BmEM-SD-1_PZ338071      -----------
Staphylococcus_arlettae_ATCC_43957_MH031694  -----------
```
